# Integrated metabolomic and transcriptomic analysis of anthocyanin metabolism in wheat pericarp

**DOI:** 10.1186/s12863-024-01294-y

**Published:** 2025-01-13

**Authors:** Jiao Wang, Lei Sun, Bo Jiao, Pu Zhao, Tianyun Xu, Sa Gu, Chenmin Huo, Jianzhou Pang, Shuo Zhou

**Affiliations:** 1 Institute of Biotechnology and Food Science, Hebei Academy of Agriculture and Forestry Sciences/Hebei Key Laboratory of Plant Genetic Engineering, Shijiazhuang, 050000 China; 2 Dryland Farming Institute of Hebei Academy of Agricultural and Forestry Sciences/Key Laboratory of Crop Drought Tolerance Research of Hebei Province, Hengshui, 053000 China; 3 Hebei Normal University, Shijiazhuang, 050000 China; 4 Hebei University of Economics and Business, Shijiazhuang, 050000 China

**Keywords:** Wheat pericarp, Anthocyanin, Metabolomic, Transcriptomic, Hormone

## Abstract

**Background:**

Wheat seeds display different colors due to the types and contents of anthocyanins, which is closely related to anthocyanin metabolism. In this study, a transcriptomic and metabolomic analysis between white and purple color wheat pericarp aimed to explore some key genes and metabolites involved in anthocyanin metabolism.

**Results:**

Two wheat cultivars, a white seed cultivar Shiluan02-1 and purple seed cultivar Hengzi151 were used to identify the variations in differentially expressed genes (DEGs) and differentially accumulated flavonoids (DAFs). Based on metabolomic data, 314 metabolites and 191 DAFs were identified. Chalcone, flavonol, pro-anthocyanidin and anthocyanidin were the most differentially accumulated flavonoid compounds in Hengzi151. 2610 up-regulated and 2668 down-regulated DEGs were identified according to transcriptomic data. The results showed that some structural genes in anthocyanin synthesis pathway were prominently activated in Hengzi151, such as *PAL*, *CAD*, *CHS* and so on. Transcription factors (TFs) of *MYB*, *bHLH*, *WD40* and some other TFs probably involved in regulating anthocyanin biosynthesis were identified. Some genes from hormone synthetic and signaling pathways that may participate in regulating anthocyanin biosynthesis also have been identified.

**Conclusions:**

Our results provide valuable information on the candidate genes and metabolites involved in the anthocyanin metabolism in wheat pericarp.

**Supplementary Information:**

The online version contains supplementary material available at 10.1186/s12863-024-01294-y.

## Background

Anthocyanins are a type of multifunctional and water-soluble natural pigments, which is widely present in plants. Plants display different colors from red to purple in the flowers, fruits, stems, and leaves due to the anthocyanins. The anthocyanin biosynthetic pathway is very conserved in many plant species, the corresponding genes have been identified and isolated. The pathway starts with phenylalanine, which catalyzed by phenylalanine ammonia-lyase (PAL), cinnamate 4-hydroxylase (C4H) and 4-coumarate: CoA ligase (4CL) to form 4 coumaroyl-CoA, chalcone synthase (CHS) mediated 4 coumaroyl-CoA and malonyl-CoA to synthetize naringenin chalcone. Then, naringenin chalcone is isomerized by chalcone isomerase (CHI) to naringenin and naringenin is converted into dihydrokaempferol by flavanone 3-hydroxylase (F3H), flavonoid 3′-hydroxylase (F3′H) and flavonoid 3′,5′-hydroxylase (F3′5′H) can hydroxylate dihydrokaempferol into dihydroquercetin or dihydromyricetin, respectively. Next, dihydrokaempferol, dihydroquercetin and dihydromyricetin are converted into leucoanthocyanidins by dihydroflavonol 4-reductase (DFR) and anthocyanidin synthase (ANS) catalyzed leucoanthocyanidins to anthocyanidins. Finally, glycosyltransferase (favonoid 3-O-glucosyltransferase, UFGT) enzyme adds sugar molecules to anthocyanidins. Anthocyanins undergo methylation, glycosylation and acylation modifications upon the activity of methyltransferases, glycosyltransferases and acyltransferases to form stably colored anthocyanins [1].

The structured gene’s temporal and spatial expression also are regulated by transcription factors. It has been reported that the MBW complex of R2R3-MYB, bHLH (basic-helix-loop-helix) and WD40 proteins regulate the anthocyanin biosynthetic pathway at the transcriptional expression. The MYB transcription factor family is one of the largest TF families with a highly conserved MYB domain at the N-terminus, which generally consists of one to four (R, 52 amino acid sequence) repeats. MYB TFs can be divided into four classes including 1R-MYB, R2R3-MYB, 3R-MYB and 4R-MYB according to the number of MYB repeats. MYB TFs generally regulate the anthocyanin level by forming an MBW complex with bHLH and WD40 TFs or self-regulating to regulate the expression of downstream target genes [2]. R2R3-MYB TFs with two repeats at the N-terminus are the largest classes of MYB group in plants and have been reported to promote or suppress the expression of the structural genes in the anthocyanin biosynthetic pathway and regulate anthocyanin metabolism. bHLH and MYB are crucial transcriptional regulators in the anthocyanin pathway, for example bHLH92 acts as a negative regulator of anthocyanin/proanthocyandin accumulation and influences seed dormancy in sheepgrass [3]. A R2R3-MYB TF, *SbMYB12* positively regulates baicalin biosynthesis in *Scutellaria baicalensis* Georgi [4]. WD40 proteins don’t exhibit catalytic activity and act as docking platforms involved in the regulation of both anthocyanin and proanthocyanin (PA) biosynthetic pathways [5], FcWD40-97/FcTTG1 interacts with FcMYB114, FcMYB123, and FcbHLH42 proteins to form MBW complex and regulate anthocyanin-biosynthesis in *Ficus carica* L [6].

Previous studies detected that mutations in the promoters and coding sequences of bHLH and MYB TFs may activate the downstream structural genes in anthocyanin biosynthetic pathway and ultimately result in different colors. *Gret1* (grapevine retrotransposon 1) retrotransposon inserted in the promoter of *VvmybA1* inhibits gene expression and causes a deficiency in the grape skin color [7]. A copia-like LTR retrotransposon inserted in the promoter of *PsMYB10.2* promotes the gene expression and activate the anthocyanin biosynthesis pathway [8]. In wheat (*Triticum aestivum* L.), anthocyanin exists in coleoptile, leaf, glume, stem, anther, and pericarp. Wheat seeds display white, red, blue, and purple colors due to various colors of wheat pericarp. Previous studies have demonstrated that MYB and bHLH TFs are the relevant anthocyanin regulatory genes, in purple pericarps of wheat seed, *Pp-D1* on chromosome 7D and *Pp3* on chromosome 2 A were identified as regulators of anthocyanin biosynthesis [9, 10]. The *TaMYC1* (KJ747954) gene, encoding a MYC-like transcription factor, was identified and isolated, which was highly expressed in purple pericarps [11]. *TaMYC1* is a synonym of *Pp3*, two distinct *TaMYC1p* and *TaMYC1w* alleles were isolated from purple- and white-grained wheat and *TaMYC1p* promoter has six copies of compound cis-acting regulatory elements, but only once was present in *TaMYC1w*, *TaMYC1p* was necessary but not sufficient for anthocyanin pigments accumulation in the pericarp tissues [12]. Two transcription factors *TaPpm1* (purple pericarp-*MYB1*) and *TaPpb1* (purple pericarp-*bHLH1*) were characterized as anthocyanin activators in purple pericarps, TaPpm1 interact with TaPpb1 to coregulate the synthesis of anthocyanin [13].

Some reports have proved that hormones also participate in regulating the anthocyanin biosynthesis. Auxin plays an important role in regulating plant growth and development, auxin can inhibit the accumulation of anthocyanin by degrading MdIAA26 protein in apple [14]. Anthocyanin biosynthesis is also induced by cytokinin, which is regulated by MYB transcription factors. Gibberellic acid can promote the transcription and mRNA accumulation of chalcone synthase (*CHS*) and corolla pigmentation in an in vitro flower bud culture system [15]. DELLA proteins in GA signaling pathway promote anthocyanin biosynthesis by sequestering MYBL2 and JAZ, which are the suppressors of the MYB/bHLH/WD40 complex in *Arabidopsis thaliana* [16]. DELLA proteins interacted with PAP1 and enhanced the transcriptional activity of PAP1 on anthocyanin biosynthetic gene expressions to positively regulate nitrogen deficiency-induced anthocyanin accumulation [17]. Ethylene signaling negatively regulates anthocyanin biosynthesis induced by sugar and light signaling [18], it has been reported that PyMYB114 activated the transcription of *PyERF4.1*/*PyERF4.2*, and PyERF4.1/PyERF4.2 interacted with PyERF3 to affect the stability of the PyERF3-PyMYB114-PybHLH3 complex, thereby inhibiting the transcription of *PyANS* and repress anthocyanin biosynthesis in red-skinned pears [19]. ABA promotes the accumulation of anthocyanins in *Lycium* fruits, the developmental cues transcriptionally activate *LbNCED1*, which enhances the accumulation of ABA, ABA stimulates the transcription of the MYB-bHLH-WD40 complex, thus upregulate the expressions of structural genes in the flavonoid biosynthetic pathway and promote anthocyanin production and fruit coloration [20]. Jasmonates (JAs) have been shown to regulate plant development, mediate defense responses, and induce anthocyanin biosynthesis in several plant species. F-box protein CORONATINE INSENSITIVE 1 (COI1), a critical regulator in JA signaling pathway and involved in multiple JA-related processes, was reported to regulate the expression of *PAP1*, *PAP2*, and *GL3*, which mediates the ‘late’ anthocyanin biosynthetic genes of *DFR*, *LDOX*, and *UF3GT*, thereby modulating JA-induced anthocyanin biosynthesis in *Arabidopsis* [21]. *AaCOI1* from *Artemisia annua* L. is involved in development, defense, and anthocyanin synthesis [22]. Jasmonate ZIM-domain (JAZ) proteins, substrates of the COI1-based SCF^COI1^ complex, interact with bHLH (Transparent Testa8, Glabra3 [GL3], Enhancer of Glabra3 [EGL3]) and R2R3 MYB (MYB75 and Glabra1) transcription factors of WD-repeat/bHLH/MYB complexes to repress JA-regulated anthocyanin accumulation and trichome initiation in *Arabidopsis thaliana* [23]. Brassinosteroid (BR) can enhances anthocyanin accumulation induced by JA in *Arabidopsis* Seedlings [24]. The grape ripening, anthocyanins and other phenolics contents and antioxidant capacity can be significantly promoted by using exogenous 24-Epibrassinolide (EBR) treatment in the grape skin [25].

In recent years, many studies on anthocyanin metabolism have been conducted using combined analysis of the transcriptome and metabolome in plants [26–28]. Here, we explored the metabolome and transcriptome of wheat pericarp in Shiluan02-1 and Hengzi151. DEGs and DEGs from the flavonoid biosynthetic pathway, hormone biosynthesis and transduction pathway, the MYB, bHLH and WD40 families were identified by transcriptome analysis. DAFs in wheat pericarp were also identified by metabolome analysis. Furthermore, we conducted the research on the regulatory networks of flavonoid biosynthesis in wheat pericarp. The findings of this study will not only provide candidate genes for breeding of wheat but also valuable flavonoid metabolic information of wheat pericarp.

## Materials and methods

### Plant materials

The seeds of Shiluan02-1 were provided by Zhanjing Huang of Hebei Normal University. Hengzi151 seeds kept in our lab is the offspring of the wheat strain Heng09guan138 and Shiluan02-1. The seeds were kept in our laboratory. Planted the seeds in the soil and germinated for 48 h in dark, then transferred the seedlings into an illumination incubator at 8 °C under long-light conditions (LL, 16 h/8 h) for 30 days. Transplanted the seedlings in greenhouse and collected the pericarp after pollination for 26 days. We collected 1.6 g Shiluan02-1 and 2.3 g Hengzi151 pericarp samples including 3 biological replicates for metabolome analysis, the samples were quickly frozen in liquid nitrogen and stored at −80 °C until use.

### RNA extraction, library construction and sequencing

Total RNA was further extracted using RNA prep Pure Plant Kit (Tiangen, Beijing, China). The RNA quality was assessed by RNA Nano 6000 Assay Kit of the Bioanalyzer 2100 system (Agilent Technologies, CA, USA). mRNA was extracted from total RNA by VAHTS mRNA Capture Beads (Vazyme Biotech, Nanjing, China). The purified mRNA was used to generate the RNA-seq library with the NEBNext^®^ UltraTM RNA Library Prep Kit (NEB, USA) and then sequenced on an Illumina HiSeq platform (Illumina, USA).

### Transcriptomic data analysis

The adaptor-containing sequences, poly-N, and low-quality reads were filtered by FASTP v0.23 to obtain high-quality reads [29]. Clean reads were further mapped to the reference genome of Triticum aestivum cv. Chinese Spring (IWGSC RefSeq v2.1) from NCBI (https://www.ncbi.nlm.nih.gov/datasets/genome/?taxon=4565) using Hisat2 v2.2.1 [30]. R package FeatureCounts v1.5.0 [31] was used to count the reads numbers mapped to each gene, each gene expression level was normalized as fragments per kilobase of transcript per million mapped reads (FPKM). The unsupervised principal component analysis (PCA) was performed by the R package PCAtools v2.8.0 [32] and the correlation analysis based on the spearman’s correlation coefficient method was performed by the function cor within R.

R package DESeq2 v1.20.0 [33] was used to identify the differentially expressed genes (DEGs) between two samples. *P*-value was adjusted by Benjamini and Hochberg method. Genes with adjusted *p*-value (padj) < 0.05 and absolute log2fold change > 2 were considered as DEGs. Plant transcription factors were predicted by iTAK v1.5 [34], the full-length protein sequences were used as queries. The genes in each TF family were presented as heatmaps.

Genes were annotated by GO, KEGG and Mercator4 databases separately. GO functional annotations were performed using local functional annotations database (http://eggnog6.embl.de/download/emapperdb-5.0.2/) by eggNOG-Mapper V2 [35]. The results of gene annotations assigned by Mercator4 were used for counting the genes participating in flavonoid and hormone metabolism, the results were visualized by mapman [36]. GO and KEGG pathway enrichment analysis of DEGs were implemented by R package clusterProfiler [37], padj < 0.05 was considered as the threshold.

### Extraction and quantification of flavonoids

The metabolites were extracted from the pericarps with three biological repeats. The samples were freeze-dried by vacuum freeze-dryer (Scientz-100 F) and crushed using a mixer mill (MM 400, Retsch). Each sample (100 mg) was dissolved in 1.2 mL 70% methanol solution and stored at 4 °C overnight, then centrifuge at 12,000 rpm for 10 min. The extracts were filtrated using a nylon syringe filter (SCAA-104, 0.22 μm pore size; ANPEL, Shanghai, China, http://www.anpel.com.cn/) and analyzed by LC/MS.

The extracts were analyzed using an UPLC-ESI-MS/MS system (UPLC, SHIMADZU Nexera X2, https://www.shimadzu.com.cn/; MS, Applied Biosystems 4500 Q TRAP, https://www.thermofisher.cn/cn/zh/home/brands/applied-biosystems.html). Chromatographic separation was conducted on an Agilent SB-C18 column (1.8 μm, 2.1 mm * 100 mm, Agilent Technologies, Santa Clara, CA, USA). LIT and triple quadrupole (QQQ) scans were obtained on a triple quadrupole-linear ion trap mass spectrometer (Q TRAP) and an AB4500 Q TRAP UPLC/MS/MS System. Positive and negative ion mode was controlled by Analyst 1.6.1 software (AB Sciex) via an ESI Turbo Ion-Spray interface.

We performed the qualitative metabolite analysis and the results of metabolome data were identified in Metware (Metware Biotechnology Co. Ltd., Wuhan, China), which were based on the secondary spectral information. The isotopic signals and repeated signals containing K^+^, Na^+^, NH4^+^ and other high-MW debris ions were simultaneously removed.

### Metabolomic data analysis

The differential metabolites were identified based on the combination of variable influence on projection (VIP) obtained from OPLS-DA model by the R package ropls [38] and *p*-value calculated by Student’s t-test. Metabolites conforming to the threshold VIP > 1.0 and *p* < 0.05 were taken as differential metabolites. The hierarchical cluster analysis of metabolites was carried out and presented as heatmaps by the R package pheatmap [39].

### Integrated metabolomic and transcriptomic analysis

Nine-quadrant associate analysis was conducted based on the combined transcriptome and metabolome data. Genes and Metabolites were annotated and mapped to the KEGG Pathway [40], which were downloaded in xml files from the KEGG Markup Language (KGML) database parsed by the R package KEGGgraph [41]. The relationships were visualized by cytoscape 3.9 [42]. O2PLS was conducted by the R package OmicsPLS [43]. Plots were drawn using the R package ggplot2 [44].

### qRTPCR analysis

Nine DEGs involved in anthocyanin biosynthesis were selected for analysis by qRT-PCR. The total RNA was extracted from wheat pericarp using the FastPure Plant Total RNA Isolation Kit (Polysaccharides&Polyphenolics-rich) (Vazyme Biotech, Nanjing, China), the cDNA was obtained by reverse transcription of the total RNA according to the HiScript III RT SuperMix for qPCR (+ gDNA wiper) (Vazyme Biotech, Nanjing, China). qRT-PCR was performed with ChamQ Universal SYBR qPCR Master Mix (Vazyme Biotech, Nanjing, China) according to the manufacturer’s instructions in a Real-Time PCR apparatus (ABI7500). The wheat actin gene was used as internal control. Quantify the gene expression level used the comparative 2^−ΔΔCT^ method and the graphs were made by GraphPad Prism 7 software. The primers used in qRT-PCR are listed in Table S6.

## Results

### Flavonoid metabolites profiles of Shiluan02-1 and Hengzi151

**Fig. 1:**
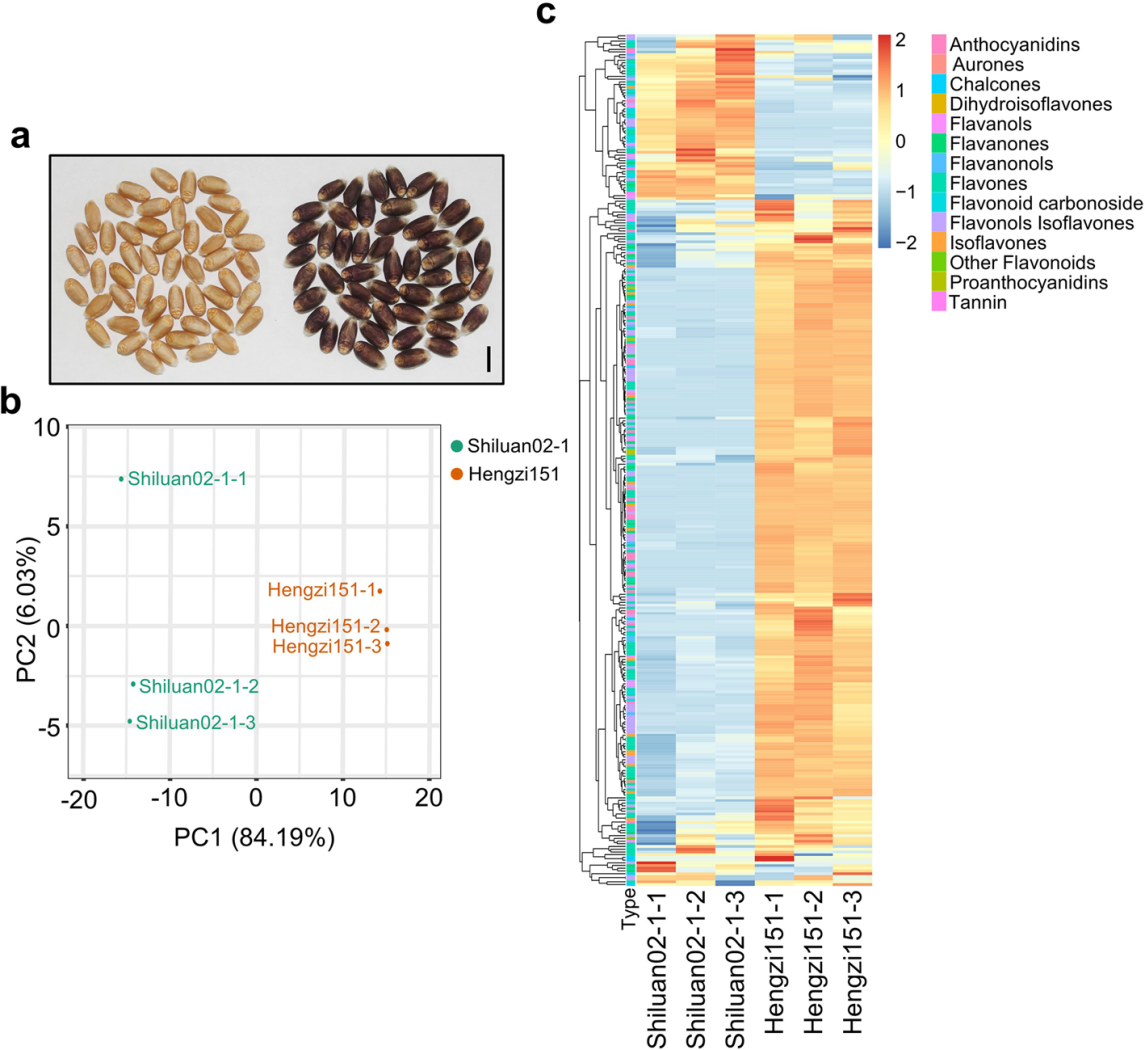
Morphological characteristics of Shiluan02-1 and Hengzi151 seeds and profiles of metabolomics. **a** The seeds of Shiluan02-1 (left) and Hengzi151 (right), the scale bar is 0.5 cm. **b** PCA of metabolome data. The X-axis represents principal component 1 (PC1), the Y-axis represents principal component 2 (PC2), different samples are distinguished by different colors. **c** Hierarchical cluster analysis (HCA) based on the relative content of flavonoid compounds detected in the flavonoid pathway

We identified and quantified the flavonoid metabolites in Shiluan02-1 and Hengzi151 (Fig. 1a), 305 flavonoids and 9 tannins were identified (Table S1). Correlation analysis according to metabolome and transcriptome data indicated the good repeatability, the correlation values within the same material were over 95% (Fig. S1). The upper right corner represents the correlation of the transcriptomes between different samples, the upper left corner stands for metabolomes. It is evident that the correlations within the same material are consistently high, over 95%, both for transcriptomes and metabolomes. The metabolite profiles were then subjected to principal component analysis, and the PCA score plots exhibited an obvious separation between Hengzi151 and Shiluan02-1. The first principal component (PC1) was 84.19% and the second principal component (PC2) were 6.03%, flavonoid metabolites present in the Hengzi151 were separated from Shiluan02-1 (Fig. 1b), indicating that the flavonoid metabolites were different between Hengzi151 and Shiluan02-1. The 314 flavonoid metabolites were classified into 14 categories, which include aurone, dihydroisoflavone, flavanonol, flavanol, isoflavone, flavanone, flavonoid carbonoside, flavonol, favone, chalcone, proanthocyanidins, anthocyanidins, tannins and other flavonoids. Hierarchical cluster analysis (HCA) was performed on the accumulation pattern of metabolites in Shiluan02-1 and Hengzi151 with 3 replicates of each sample, which showed the most of metabolite levels were higher in Hengzi151, while a few metabolite levels were higher in Shiluan02-1 (Fig. 1c).

**Fig. 2:**
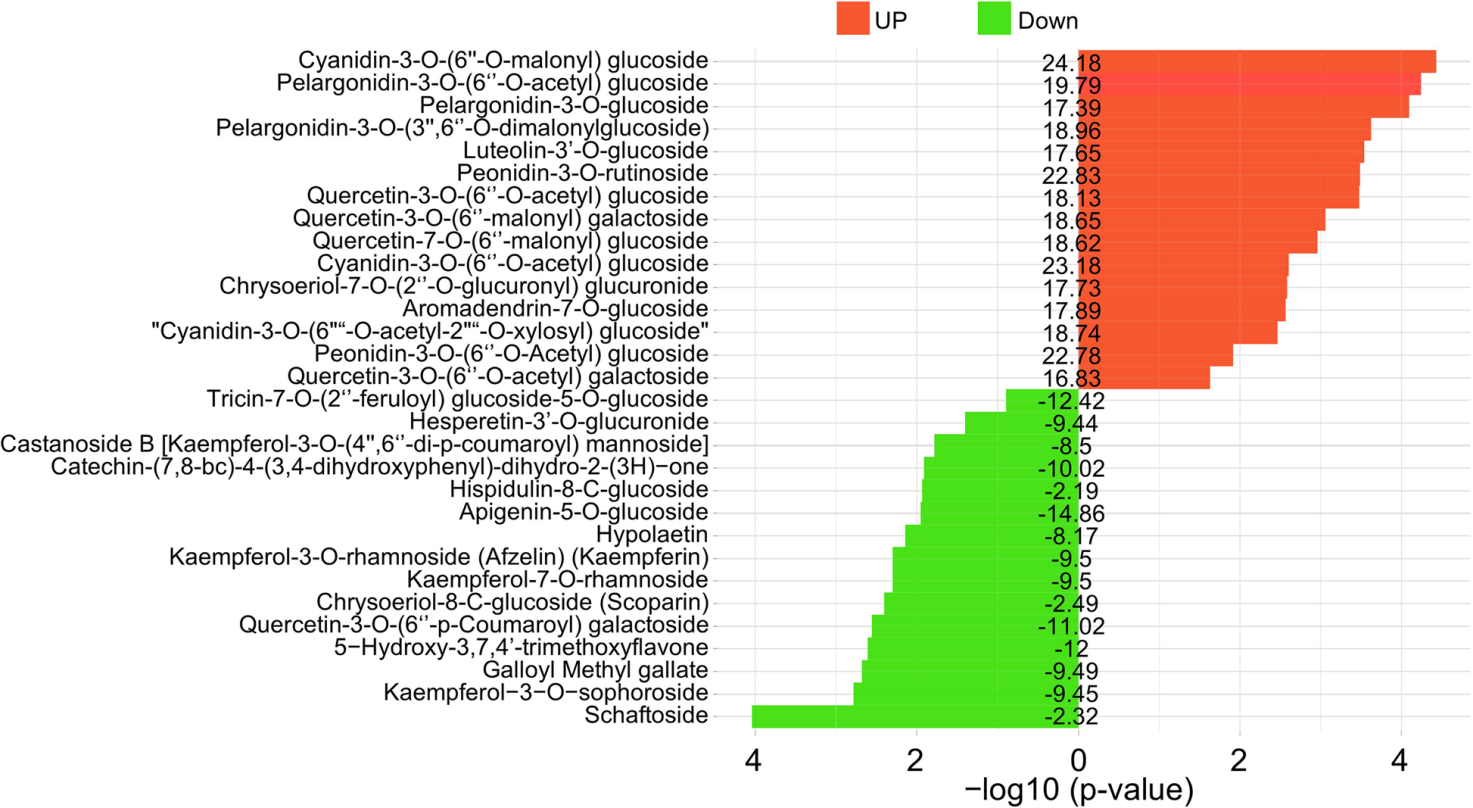
The top 15 up/down-regulated differentially accumulated flavonoids (DAFs) in Hengzi151. Numbers labeled in the middle represent the expression level, the barplot stands for -log10 (*p*-value) of each DAF; red bars indicate up-regulated flavonoid compounds, green bars indicate down-regulated flavonoid compounds

One hundred and ninety-one flavonoid metabolites (60.83% of the total, including 161 up-regulated and 30 down-regulated) were differentially expressed among the 314 flavonoid metabolites with the absolute Log2FC ≥ 1 and VIP value ≥ 1. Dihydroisoflavone, proanthocyanidin, tannin, chalcone, flavanonol, flavanol, isoflavone, flavanone, flavonoid carbonoside, anthocyanidin, flavonol and favone were included (Table S2). Among them, cyanidin-3-O-(6’’-O-malonyl) glucoside and apigenin-5-O-glucoside were identified as the most significantly up- and down-regulated differentially accumulated flavonoids in Hengzi151, respectively (Fig. 2). Metabolites involved in taes00941 (flavonoid biosynthetic pathway), taes00942 (anthocyanin biosynthetic pathway) and taes00944 (flavone and flavonol biosynthetic pathway) pathways were also identified, the most of metabolites involved in flavonoid synthetic pathway and flavone and flavonol biosynthetic pathway were up-regulated in Hengzi151 (Fig. S2a and S2c). While all metabolites in anthocyanin biosynthetic pathway were the derivatives of cyanidin and pelargonidin, they were the major anthocyanins in the purple wheat seed pericarp (Fig. S2b). The rich accumulation of cyanidin and pelargonidin derivatives in the seeds of purple wheat makes that beneficial on human health. Therefore, Hengzi151 can be used for functional food production and in breeding anthocyanin enriched wheat.

### Transcriptome analysis in pericarp of Shiluan02-1 and Hengzi151

To investigate the transcriptomic changes between Shiluan02-1 and Hengzi151 pericarp, we generated the transcriptome analysis. Three biological replicates of each sample were collected for RNA-seq assays. A total of 408,178,468 high-quality (Q30 > 93%) clean reads were obtained, with an average of 68,029,744 reads (> 8 Gb) for biological replicates. The reads were mapped to the reference genome of CS (IWGSC RefSeq v2.1) and the average unique alignment rate was 82.61% (Table 1).

**Table 1:** Sequencing data statistics of 6 samples

Sample	Total Reads	Clean Reads	Bases (bp)	Q20 (%)	Q30 (%)	Total mapped (%)	Uniquely mapped (%)
Shiluan02-1-1	71,421,336	67,298,550	10,713,200,400	97.67	94.1	94.45	83.35
Shiluan02-1–2	68,013,004	64,087,232	10,201,950,600	97.58	93.91	94.31	83.18
Shiluan02-1–3	70,282,716	66,424,242	10,542,407,400	97.55	93.73	94.20	82.55
Hengzi151-1	78,039,464	73,548,706	11,705,919,600	97.58	93.95	93.29	82.21
Hengzi151-2	71,814,762	67,708,204	10,772,214,300	97.67	94.08	93.27	82.12
Hengzi151-3	73,251,670	69,111,534	10,987,750,500	97.59	93.96	93.24	82.25
Total	432,822,952	408,178,468	64,923,442,800				

**Fig. 3:**
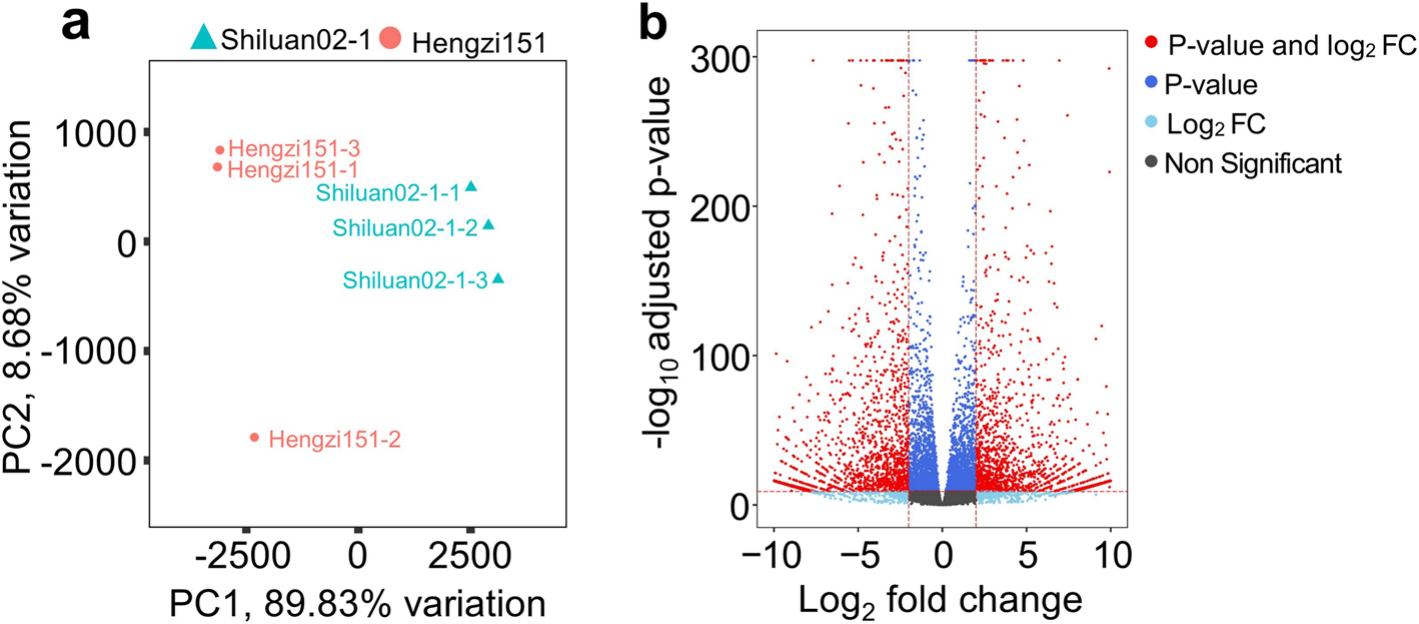
Profiles of transcriptomics. **a** PCA of transcriptome data. The X-axis represents PC1, the Y-axis represents PC2, different samples are distinguished by different colors. **b** Volcano plots of DEGs in the comparison of Hengzi151 and Shiluan02-1, red dots indicate genes with the *p*-value (padj) < 0.05 and log2fold change > 2

PCA analysis was performed on Shiluan02-1 and Hengzi151, PC1 and PC2 were 89.83% and 8.66% separately (Fig. 3a). Transcriptomic expression profiling of all the samples revealed the expressed genes, 5,279 DEGs were identified between two samples, including 2,610 upregulated genes and 2,669 downregulated genes with the filter criteria |Log2FC| ≥2 and FDR < 0.05 (Fig. 3b).

**Fig. 4:**
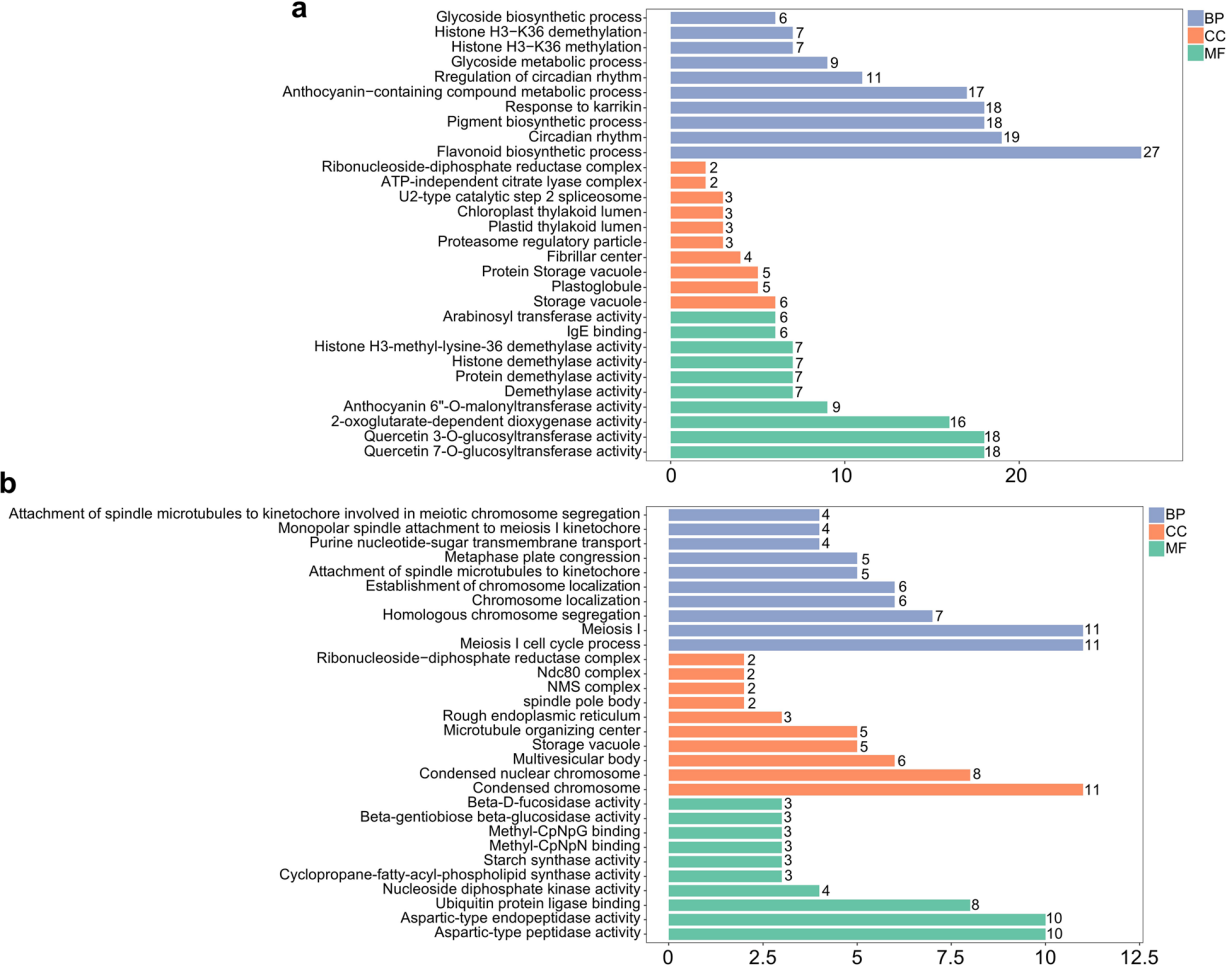
GO classifications of DEGs according to three GO categories: biological process (BP), cellular component (CC) and molecular function (MF). **a** GO classifications of up-regulated DEGs. **b** GO classifications of down-regulated DEGs. Numbers of genes enriched in each catogory are annotated near the bar

GO enrichment analysis of DEGs involved in three major GO categories: biological process (BP), cellular component (CC) and molecular function (MF) were conducted. For up-regulated DEGs, 27 flavonoid biosynthetic process, 18 pigment biosynthetic process and 17 anthocyanin-containing compound metabolic process were identified in the BP category; 6 storage vacuole, 5 plastoglobule and 5 protein storage vacuole were enriched in CC category; 18 quercetin 3-O-glucosyltransferase activity, 18 quercetin 7-O-glucosyltransferase activity and 9 anthocyanin 6”-O-malonyltransferase activity were enriched in MF category (Fig. 4a). As to down-regulated DEGs, meiosis I process in BP category, condensed chromosome in CC category, and aspartic-type peptidase activity in MF category were the most enriched (Fig. 4b).

**Fig. 5:**
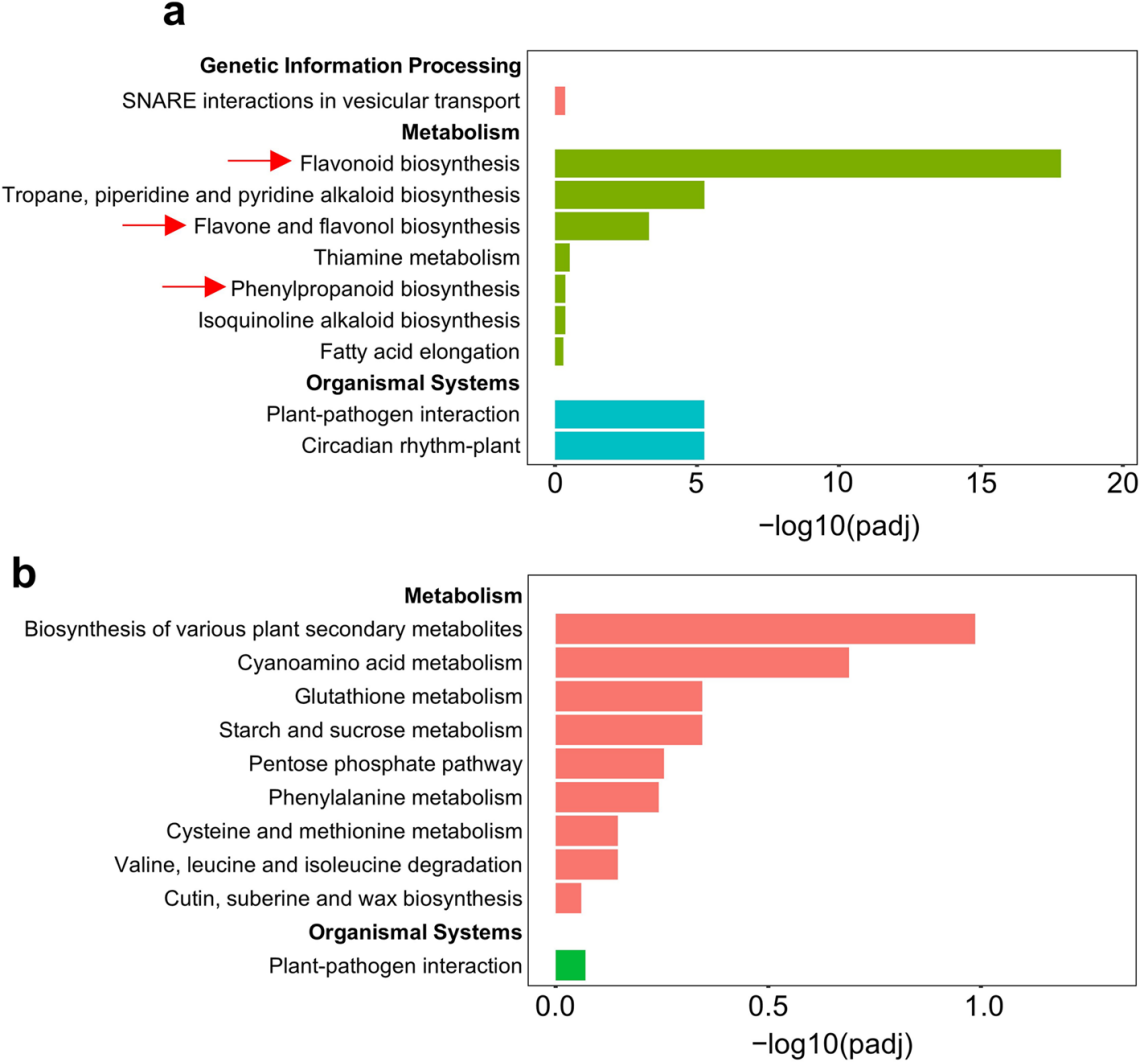
Significantly enriched KEGG pathways of up-regulated **(a)** and down-regulated **(b)** DEGs between Hengzi151 and Shiluan02-1. Enriched pathways are classified based on the six main categories of KEGG pathway database. X-axis means the -log10 (*p*-adj) value, Y-axis means the KEGG pathways

KEGG analysis was performed to explore the biological functions and gene interactions. For up-regulated DEGs, were enriched in 33 flavonoid biosynthesis pathways, 15 tropane, piperidine and pyridine alkaloid biosynthesis pathways, 5 flavone and flavonol biosynthesis pathways and 22 phenylpropanoid biosynthesis pathways were enriched in metabolic pathway, 5 SNARE interactions in vesicular transport in genetic information processing, 44 plant-pathogen interaction and 17 circadian rhythm-plant in organismal systems (Fig. 5a). As to down-regulated DEGs, they were the most enriched in biosynthesis of various plant secondary metabolites, cyanoamino acid metabolism, glutathione metabolism and starch and sucrose metabolism in metabolic pathway (Fig. 5b).

**Fig. 6:**
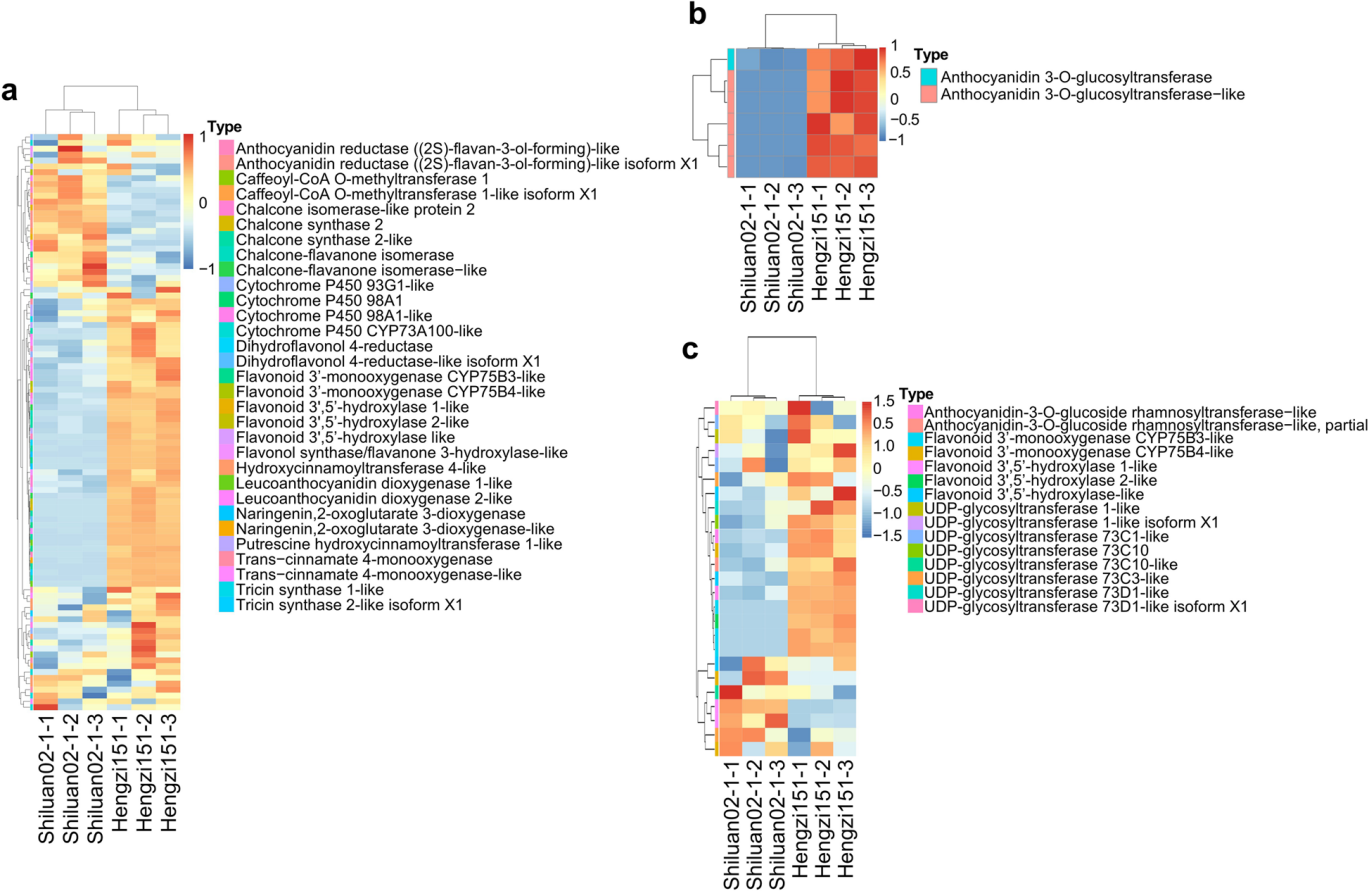
Cluster analysis of DEGs between Hengzi151 and Shiluan02-1 mapped to taes00941 (**a**), taes00942 (**b**), and taes00944 (**c**)

The DEGs were mapped to the biosynthetic pathway of taes00941, taes00942 and taes00944. There were all 129 DEGs were annotated, including 98 DEGs in the flavonoid biosynthetic pathway, which could be divided in 16 different kinds of genes (Fig. 6a); 6 DEGs in anthocyanin biosynthesis (1 anthocyanidin 3-O-glucosyltransferase gene and 5 anthocyanidin 3-O-glucosyltransferase gene-like genes) (Fig. 6b); 25 DEGs in the flavone and flavonol biosynthetic pathway, 4 different kinds of genes included (Fig. 6c), and the most of DEGs were up-regulated.

### Combined transcriptomic and metabolomic analysis revealed the biosynthesis of anthocyanin in wheat pericarp

Transcriptomic and metabolomic data were used to understand the anthocyanin biosynthetic pathway in pericarp. Large numbers of flavonoids were detected in Hengzi151, most of flavonoid content was higher in Hengzi151. A nine-quadrant plot of DEGs and DAFs correlations based on correlation coefficient values (R^2^ > 0.9 and *P*-value < 0.05) showed that large numbers of DAFs and DEGs were present in the first and third quadrants, with a negative correlation in the first quadrant and a positive correlation in the third quadrant (Fig. S3). An integrated O2PLS analysis of the transcriptome and metabolome datasets was conducted to identify the potential differential metabolites and genes, the top 15 metabolites substantially affected the transcriptome and the top 15 genes in transcriptome strongly influenced the metabolome were listed in Fig. S4.

**Fig. 7:**
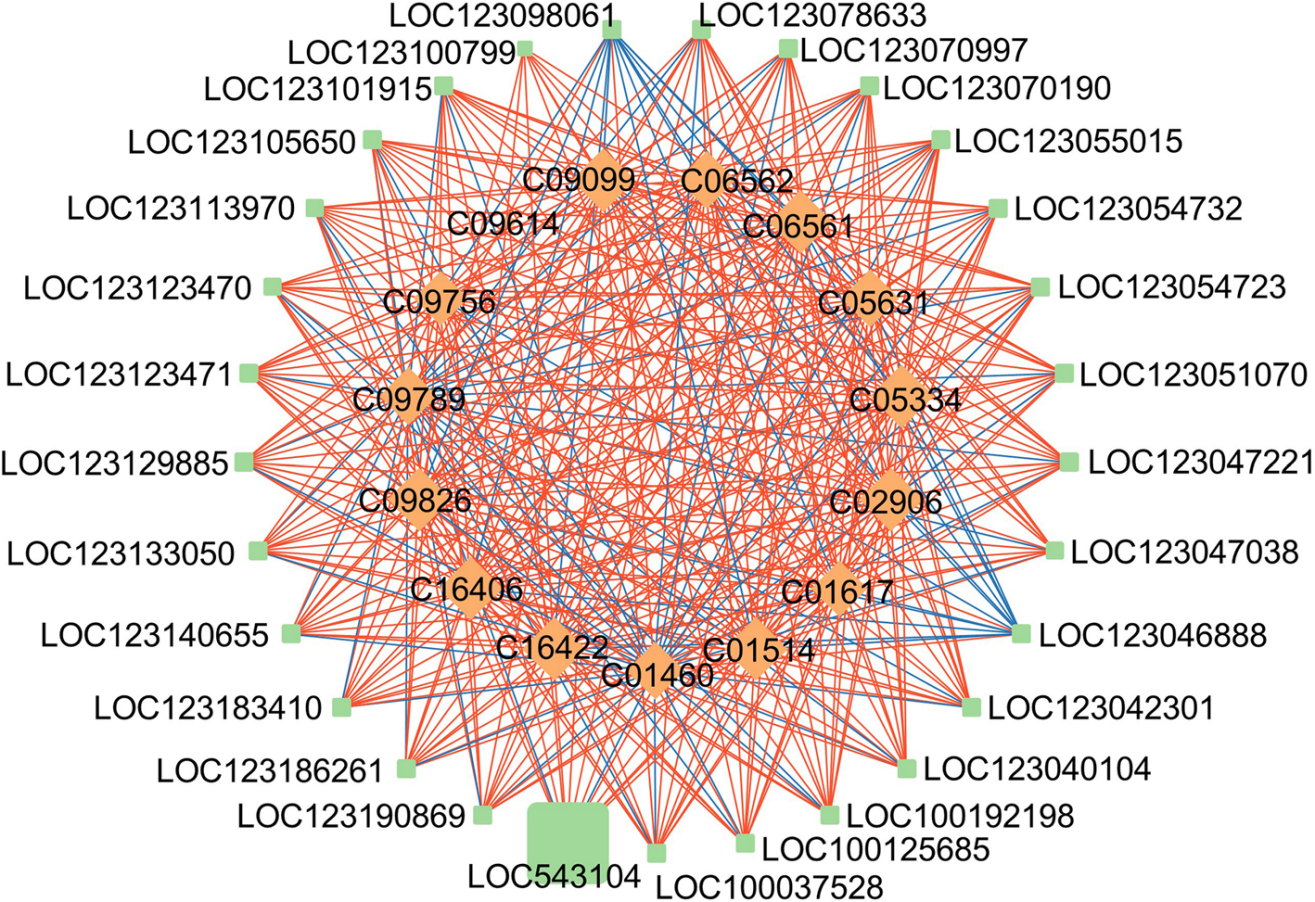
Connection network between DEGs and DAFs in Hengzi151 vs. Shiluan02-1 comparison in the taes00941. Green boxes represent genes, yellow diamonds represent metabolites. The size of green box and yellow diamond represents the number of associations between the metabolites and genes, respectively. Red lines indicate positive correlation, blue lines indicate negative correlation

 To better understand the regulatory network of flavonoid metabolism process, the DEGs and DAMs involved in flavonoid biosynthetic pathway (taes00941), anthocyanin biosynthetic pathway (taes00942) and flavone and flavonol biosynthetic pathway (taes00944) were further screened based on KEGG database. Pearson correlation coefficient analysis was performed. DEGs and DAMs were filtered by strong correlation coefficient values (R^2^ > 0.9 and *P*-value < 0.05). Red lines mean positive correlation while blue lines indicate negative correlation. The node size represents the degree of interactions. The results indicated that 29 structural genes and15 flavonoid metabolites were simultaneously mapped to taes00941pathway, LOC543104-chalcone synthase 2 was the most correlated gene (Fig. 7); 6 genes and 10 metabolites had high correlation coefficient in taes00942 pathway, LOC123179872 -anthocyanidin 3-O-glucosyltransferase-like was the most correlated gene (Fig. S5a); 7 genes and 15 metabolites were highly correlated in taes00944 pathway, LOC123047038-flavonoid 3’,5’-hydroxylase 2-like- and C11620 (Syringetin) were the most correlated gene and metabolite (Fig. S5b).

### Regulatory network of anthocyanin biosynthesis

**Fig. 8:**
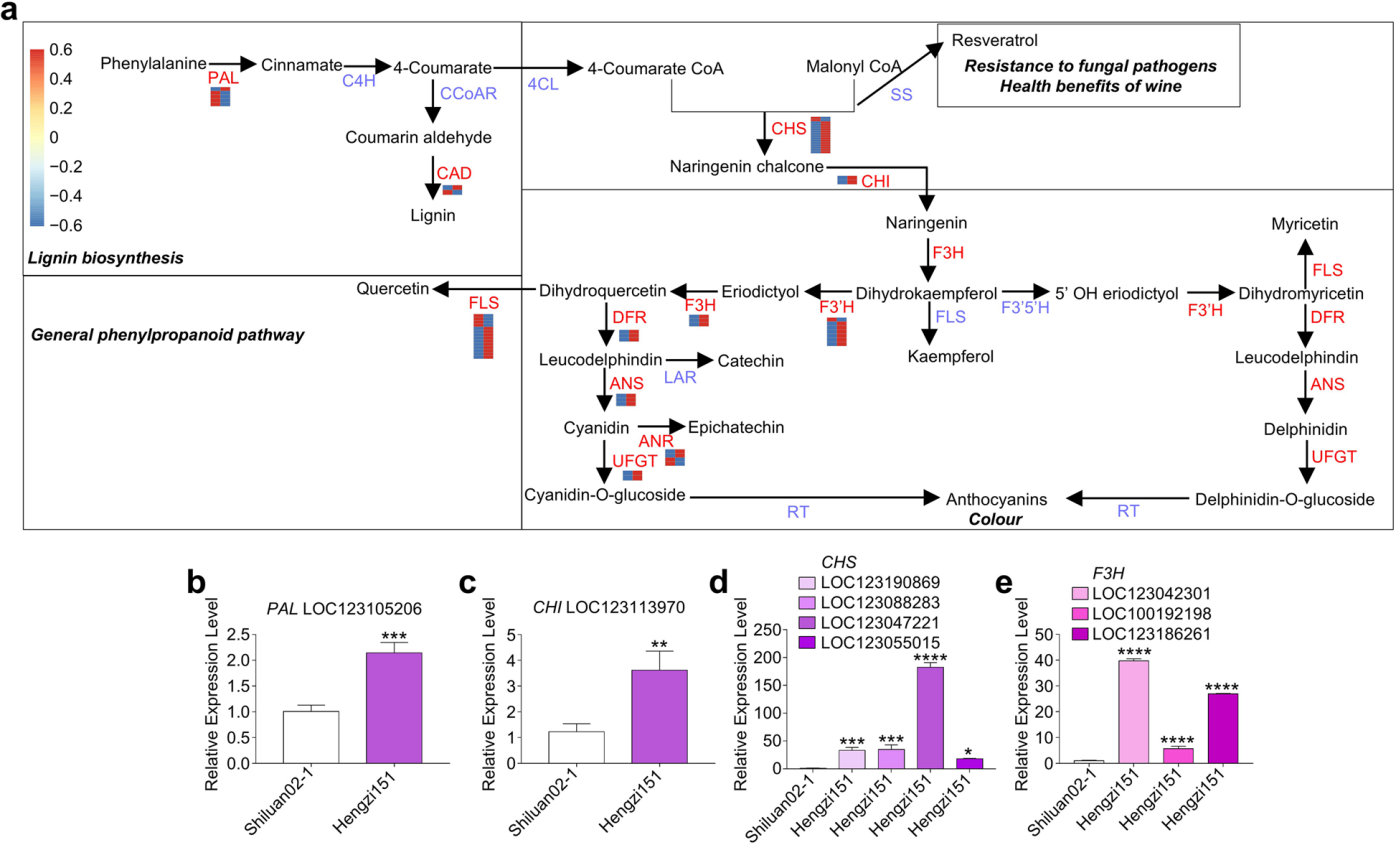
Biosynthetic pathway of anthocyanin biosynthesis and the qRT-PCR verified the DEGs related to anthocyanin biosynthesis. **a** Key genes and the heatmap of DEGs involved in biosynthesis pathway, The catalyzed enzymes are marked with blue color, the abbreviations of enzymes are as follows: PAL (phenylalanine ammonia lyase), C4H (cinnamate 4-hydroxylase), CHS (chalcone synthase), CCoAR (cinnamoyl-CoA reductase), CAD (cinnamyl alcohol dehydrogenase), 4CL (4-coumarate: CoA ligase), CHS (chalcone synthase), FLS (flavonol synthase), DFR (dihydroflavonol 4-reductase), ANS (leucoanthocyanidin dioxygenase), UFGT (UDP-glycosyltransferase), F3H (flavanone 3-hydroxylase), F3′H (flavonoid 3′-hydroxylase), F3′5′H (flavonoid 3′,5′-hydroxylase), ANR (anthocyanidin reductase). **b**-**e** The qRT-PCR verified the DEGs related to anthocyanin biosynthesis, relative expression levels of qRT-PCR were calculated using actin as a standard. Error bars represent mean ± standard deviation (*n* = 3). * indicates *p* < 0.05, ** indicates *p* < 0.01, *** indicates *p* < 0.001, **** indicates *p* < 0.0001

 To better understand the regulatory network of flavonoid metabolism process, the correlation analysis between DEGs and metabolites annotated in the flavonoid metabolism pathway was performed. Based on the MapMan annotation together with KEGG analysis results, the DEGs related to flavonoids were focused on, among which there were 61 co-expressed DEGs. 61 structural genes showed higher correlation coefficient values (R^2^ > 0.9 and *P*-value < 0.05) with 8 flavonoids. The 61 structural genes contain 5 *PAL*, 2 *CAD*, 16 *CHS*, 2 *CHI*, 14 *FLS*, 3 *F3H*, 7 *F3*′*H*, 3 *DFR*, 3 *ANS*, 4 *ANR* and 2 *UFGT* genes, which are the major structural genes in the anthocyanin metabolism pathway (Fig. 8). These genes were identified to be involved in the biosynthesis of anthocyanin and have important roles in regulating plant color. Regulating the expression of these key enzymes through genetic engineering can effectively change the color of plants, which is of great significance for agricultural breeding. To validate transcriptome results, we further selected 9 structural genes (1 *PAL*, 1 *CHI*, 4 *CHS* and 3 *F3H*) to analyze their expressions by qRT-PCR. As shown in Fig. 8b-e, the expressions of all selected genes were upregulated in Hengzi151, which were highly consistent with the transcriptome results. The high expressions of structural genes increased the synthesis and content of anthocyanins, which finally makes the seed coat purple.

### TFs involved in anthocyanin biosynthesis

**Fig. 9:**
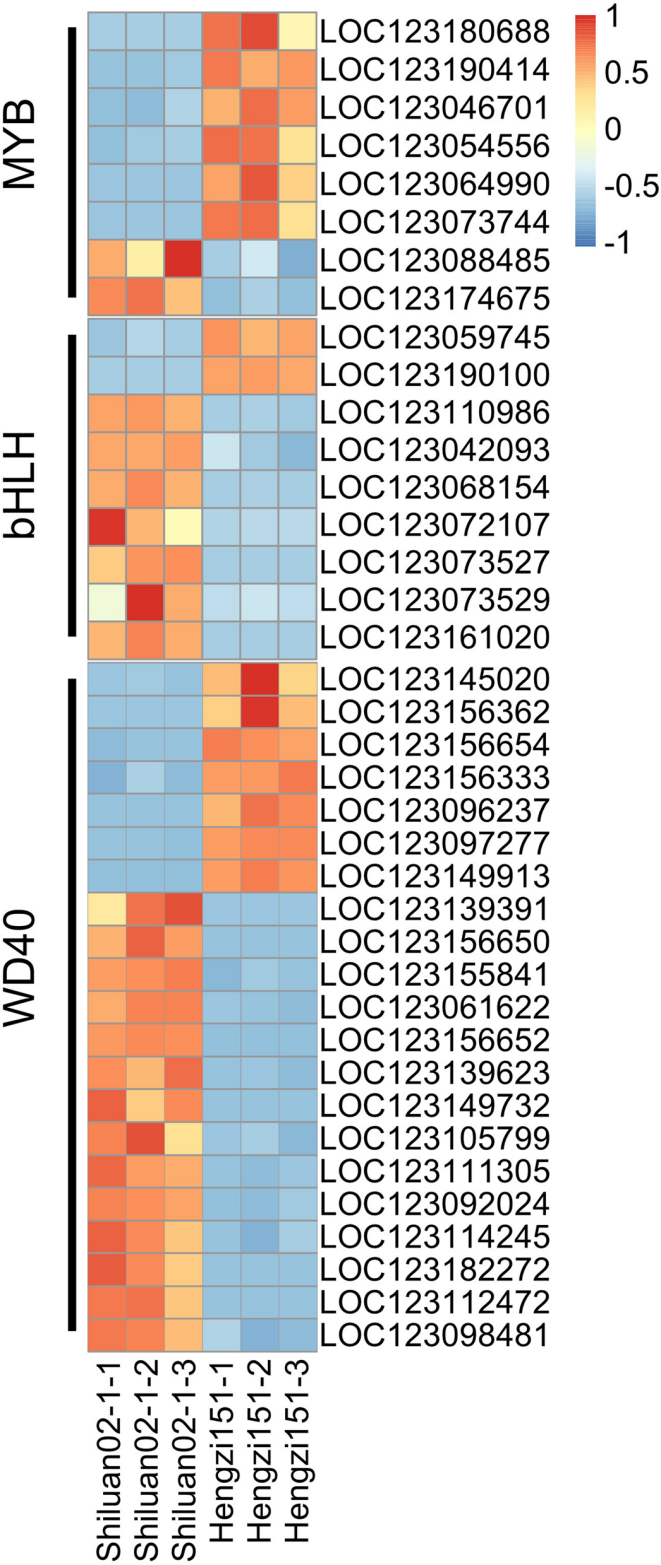
The differentially expressed MYB, bHLH and WD40 transcription factor genes identified between Hengzi151 and Shiluan02-1. The expression level was displayed based on scaled FPKM values

 The anthocyanin biosynthesis was regulated by MBW complex, previous reports indicated that *TaPpb1/TaMYC1* highly expressed, together with *TaPpm1* to regulate anthocyanin biosynthesis in wheat purple pericarp [13]. Thus, some *MYB*, *bHLH* and *WD40* transcription factors considered as relevant anthocyanin regulatory genes were identified according to the transcriptomic results, 8 *MYB* (6 up-regulated and 2 down-regulated), 9 *bHLH* (2 up-regulated and 7 down up-regulated) and 21*WD40* (7 up up-regulated and 14 down up-regulated, Fig. 9) genes were obtained. Among them, the expression level of LOC123190100 encoding a MYC transcription factor in subfamily of bHLH (*TaMYC1/ TaPpb1*) was extremely highly (322.73) expressed in Hengzi151 than in Shiluan02-1 (0.010) according to the transcriptomic data (Table S3). Anthocyanin biosynthesis is also regulated by other transcription factors, including *bZip*, *MADS*-box, *WIP* and *WRKY* at the transcriptional level [45]. In this study, 10 *AP2/ERF*, 5 *B3*, 3 *bZIP*, 5 *C2H2*, 7 *C2C2*, 2 *C3H*, 2 *WRKY*, 6 *GARP*, 4 *HB*, 2 *GARS*, 6 *MADS*, 2 *HSF*, 2 *SRS*, 3 *CPP*, 23 *FAR1* and 7 *NAC* transcription factors were identified (Table S4). Among which, an ethylene-responsive transcription factor RAP2-13-like gene of LOC123154669 showed high expression in Hengzi151, while an ethylene-responsive transcription factor 3-like gene of LOC123042630 and AP2-like ethylene-responsive transcription factor BBM1 gene of LOC123138224 showed high expression in Shiluan02-1. It was reported that AP2 negatively regulated PA biosynthesis in *Arabidopsis* by activating and interacting with a key suppressor of the PA pathway MYBL2 [46]. There are also other types of transcription factors with up/down-regulated expression level, they may be involved in regulating the anthocyanin biosynthesis pathway, which can be the candidate genes.

### DEGs identified in hormone biosynthesis and signaling that may involve in anthocyanin biosynthesis

**Fig. 10:**
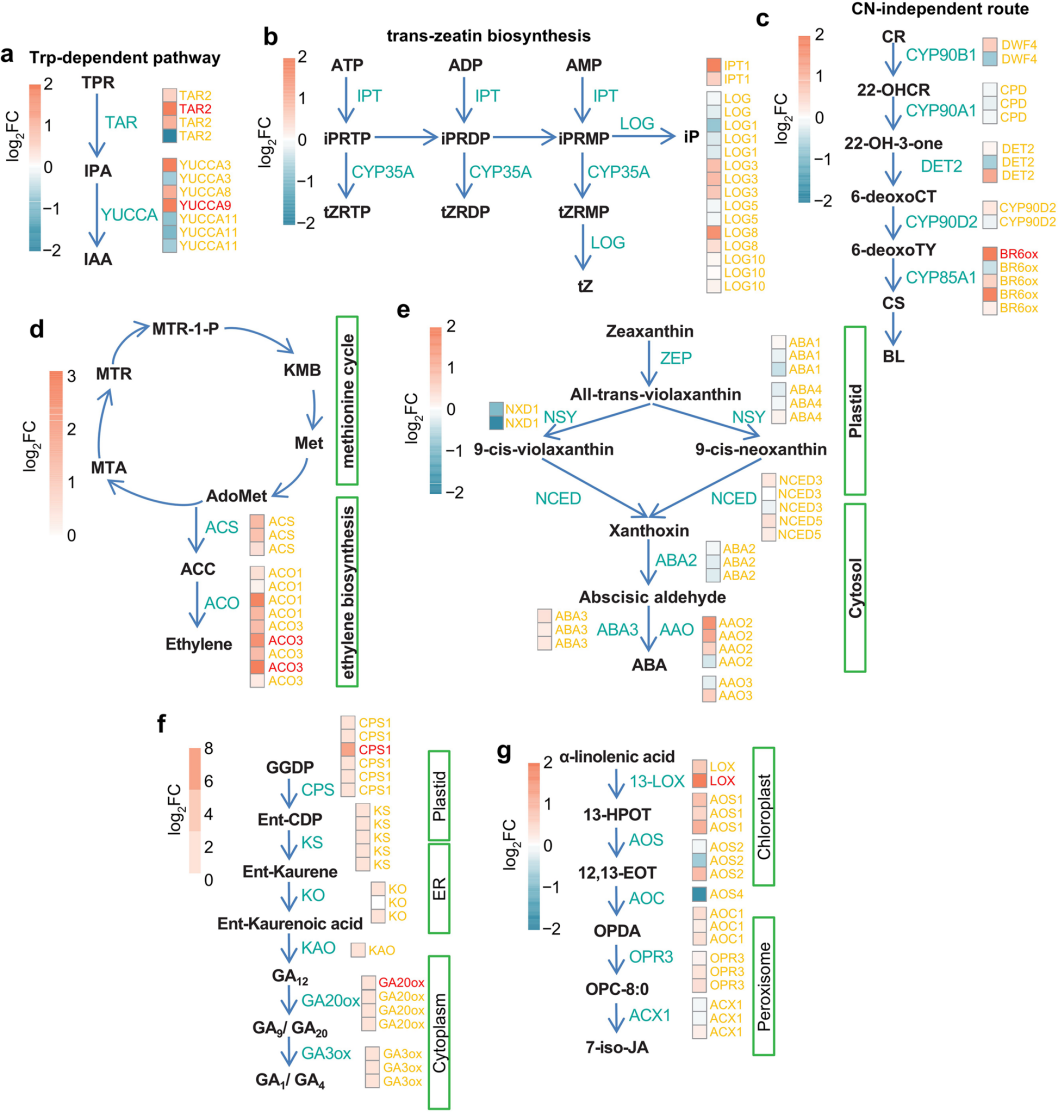
The DEGs related to auxin (**a**), cytokinin (**b**), BR (**c**), ethylene (**d**), ABA (**e**), gibberellin (**f**) and JA (**g**) biosynthesis. The precursors and intermediates involved in the biosynthesis processes are indicated in black and enzymes are marked in cyan, respectively. Gene names are shown beside their encoding products, the DEGs are colored in red and orange. Heatmaps showed the expression level of genes based on scaled FPKM values

We further analyzed the expression levels of genes participating in hormone biosynthesis and signaling pathways. All of the expressed genes were annotated by Mercator 4, which is an online tool for assigning functional annotations. Genes participating in auxin, cytokinin (CK), brassinolide (BR), ethylene, abscisic acid (ABA), gibberellin (GA) and jasmonic acid (JA) biosynthesis were filtered. The majority of the genes in cultivar Hengzi151 showed relatively higher expressions. Some genes encoding key enzymes of TAR2 (tryptophan aminotransferase related 2) and YUCCA (flavin-containing monooxygenase) regulating auxin biosynthesis were obviously up-regulated in Hengzi151 (Fig. 10a). 2 genes of LOC123144059 and LOC123175463 encoding IPT1 (adenylate isopentenyltransferase-like) and 4 LOG genes of LOC123088143, LOC123093512, LOC123098787 and LOC123149494 encoding cytokinin riboside 5-monophosphate phosphoribohydrolase showed higher expression level in CK biosynthesis in Hengzi151 (Fig. 10b). For BR and ethylene, there are also some genes identified, such as BR6ox genes (LOC123043102 and LOC123184190) (Fig. 10c), most of ACO genes had significantly high expression in Hengzi151 (Fig. 10d). The expression of genes (LOC123105343, LOC123105344, LOC123113609, LOC123123132) encoding AAO (abscisic aldehyde oxidase) in ABA biosynthesis were also up-regulated (Fig. 10e). In GA biosynthesis, the most identified genes were highly expressed (Fig. 10f). For JA synthesis, one LOX (13-lipoxygenase) gene ofLOC123136741 was identified to be up-regulated (Fig. 10g). The results indicated that plant hormones may promote the biosynthesis and work with each other to positively regulate the anthocyanin mentalism.

Genes participating in hormone signal transductions were further identified based on the KEGG pathway database. According to taes040475, 945 genes were mapped, and 48 genes were identified as DEGs. The 48 DEGs almost covered all of the signal transduction pathways and 34 genes showed a relatively higher expression in Hengzi151. But only 9 genes meet the criteria of the absolute log2 (fold change) ≥ 2 and showed significant differences, including the genes of LOC123042307 (auxin-induced protein X10A-like), LOC123058218 (auxin transporter-like protein 2), LOC123130722 (auxin-responsive protein SAUR36-like) and LOC123185943 (auxin-responsive protein SAUR72-like) participating in auxin signaling transduction. The rest genes were LOC123063562 (pseudo histidine-containing phosphotransfer protein 5-like isoform X1), LOC123190998 (two-component response regulator ORR6-like), 2 pathogenesis-related protein 1-like genes of LOC123121275, LOC123108033 and LOC123157475 (abscisic acid receptor PYL4-like) (Fig. S6). The results indicated that there is a rather complicated network of hormones regulating anthocyanin metabolism in wheat. It is very necessary to elucidate the molecular regulatory network of anthocyanins metabolism regulated by hormones, which will provide important guiding significance for the selection of anthocyanin rich varieties through molecular breeding.

## Discussion

It has been reported that flavonoid compounds are beneficial to human health. In this study, we performed metabolomic analysis to excavate some differentially accumulated flavonoid compounds in Hengzi151. According to the results, the flavonoid content of Hengzi151 was higher than that of Shiluan02-1 (Fig. 1c). There are three branches in anthocyanin pathway to produce red cyanidin, brick-red pelargonidin, and blue delphinidin pigments [1], UFGT enzyme adds sugar molecules to anthocyanidins to form cyanidin-3-glucoside, pelargonidin-3-glucoside, and delphinidin-3-glucoside. Cyanidin-3-O-glucoside that is mainly present in plants such as black rice, black beans, and purple potatoes. In our study, cyanidin-3-O-(6’’-O-malonyl) glucoside, cyanidin-3-O-(6’’-O-malonyl) glucoside, cyanidin-3-O-(6’’-O-acetyl) glucoside, peonidin-3-O-rutinoside, peonidin-3-O-(6’’-O-Acetyl) glucoside, pelargonidin-3-O-(6’’-O-acetyl) glucoside, and pelargonidin-3-O-(3’’,6’’-O-dimalonylglucoside) were the top 7 anthocyanin compounds with the highest accumulation in Hengzi151 (Fig. 2). Phloretin is a chalcone compound and highly accumulated in Hengzi151, which displays a range of pharmacological effects including antibacterial, anticancer, cellular and organ protective properties both in vitro and in vivo [47]. Cajanol, an isoflavone with antiplasmodial, antifungal and anticancer activity [48–50], showed higher accumulated in Hengzi151. Other metabolites, such as aromadendrin-7-O-glucoside (dihydroflavonoid), 2,6,7,4’-tetrahydroxyisoflavanone (dihydroisoflavone), 7-O-methyleriodictyol (flavanol), eriodictyol-8-C-glucoside (flavanones), chrysoeriol-7-O-(2’’-O-glucuronyl) glucuronide (flavone), luteolin-8-C-glucoside-7-O-glucoside (flavonoid carbonoside), and quercetin-3-O-(6’’-malonyl) galactoside (flavonol) were differentially accumulated in Hengzi151 (Table S2). Therefore, the differentially accumulated flavonoid compounds cause the seeds to appear purple colored, Hengzi151 can be used for wheat breeding.

According to the significant correlation of DEGs and DAFs, we identified some differentially expressed structural genes in the anthocyanin metabolic process, including *PAL*, *CAD*, *FLS*, *CHS*, *CHI*, *F3H*, *DFR*, *ANS*, *ANR*, *F3*′*H*, and *UFGT* genes (Fig. 8). PAL catalyzes the first step as a key and rate-limiting enzyme in general phenylpropanoid pathway, which is the first stage of anthocyanin biosynthesis. Previous studies have shown that the higher expression level of *PAL* and *4CL* resulted the production of p-coumaroyl-CoA and provided adequate precursor metabolites for flavonoid biosynthesis, which might be the key reasons for the higher concentrations of anthocyanin, flavonol and flavone in the purple tea leaves [51]. In this study, 5 *PAL* genes were identified, the transcript level of the *PAL* genes showed large differences between Shiluan02-1 and Hengzi151. The expression of one *PAL* gene (LOC123105206) was significantly higher in the Hengzi151 than in the Shiluan02-1, while other 4 genes were significantly lower in the Hengzi151 than in the Shiluan02-1 (Fig. 8). That the *PAL* gene activate the phenylpropanoid pathway and provide more primary substrates for other pathways. After some steps, anthocyanins are ultimately formed, flowers and fruit present different colors of orange, pink, red, purple, and blue due to the anthocyanins, anthocyanins benefit to human health with the antioxidant activity [52–54]. The anthocyanin pathway has three branches and form pelargonidin, cyanidin, and delphinidin anthocyanidins, which will affect the anthocyanin types and the coloration of plants, F3’H and 3’5’H are key enzymes and responsible for the biosynthesis of cyanidin- and delphinidin-based anthocyanin pigments, the relative proportion of the two types of anthocyanins is largely under genetic control and determines the color variation among red/purple/blue berry grape varieties [55]. In this study, the increasing expression level of *F3*′*H* may promote the synthesis of cyanidin and delphinidin in the Hengzi151 compared with Shiluan02-1. The genes downstream of *F3*′*H* also showed higher expression level in Hengzi151 than in Shiluan02-1, including *DFR*, *ANS*, *ANR*, and *UFGT*, which might also eventually promote the higher level of anthocyanidins in the Hengzi151 compared with the Shiluan02-1 (Fig. 8).

Anthocyanin synthesis of plants is a complex network, various genes and enzymes play a regulatory role in the pathway. In addition to MBW complex, there are also some other transcription factors regulating anthocyanin biosynthesis. For example, *TTG2* gene encoding a WRKY transcription factor, is regulated by the MBW complex in the context of trichome and pericarp development, *ttg2-1* mutant is a lack of PA flavonoid pigments in the pericarp [56, 57]. Overexpression of *MdWRKY11* promoted the expression of *F3H*, *FLS*, *DFR*, *ANS* and *UFGT* and increased the accumulation of flavonoids and anthocyanin in apple calli [58]. PyWRKY26 interact with PybHLH3 and co-target the *PyMYB114* promoter to regulate anthocyanin accumulation in red-skinned pear [59]. McWRKY71 interacted with McMYB12 and directly bound the *McANR* promoter to participate in the regulation of PA biosynthesis in *Malus* crabapple [60]. Here, we identified two WRKY genes of LOC100049024 (probable WRKY transcription factor 26) and LOC123158136 (WRKY transcription factor WRKY28-like) with down and up-regulated expression in Hengzi151, respectively. Previous studies have proved that bZIP transcription factor HY5 (ELONGATED HYPOCOTYL5), a central positive regulator of light signaling pathways in plants, also participate in regulating anthocyanin biosynthesis, PIF3 regulates anthocyanin biosynthesis in an HY5-dependent manner with both factors directly binding anthocyanin biosynthetic gene promoters in *Arabidopsis* [61], HY5 directly bind to G- and ACE-boxes in the promoter region of *PAP1* and activate the expression of *PAP1* to regulate the anthocyanin biosynthesis [62]. MdHY5 promoted anthocyanin accumulation by regulating expression of the *MdMYB10* gene and downstream anthocyanin biosynthesis genes [63], PyHY5 bonded to the G-box motifs of PyMYB10 and PyWD40 promoters of to enhance the gene expression, and promoted anthocyanin accumulation in red ‘Yunhongli No. 1’ [64]. A *HY5*-like gene (LOC123127245), found in our transcriptome data, was significantly up-regulated in Hengzi151, which may regulate the anthocyanin biosynthesis. NAC transcription factors are also involved in anthocyanin biosynthesis [65], such as MdNAC52 in apple regulates biosynthesis of anthocyanin and proanthocyanidin through MdMYB9 and MdMYB11 [66], MdNAC42 interacted with MdMYB10 to regulate anthocyanin accumulation in red-fleshed apple [67], *ANAC078* in *Arabidopsis* regulates flavonoid biosynthesis [68]. Here, we identified 7 *NAC* transcription factors (3 down-regulated and 4 up-regulated in Hengzi151), which may involve in the anthocyanin biosynthesis in purple pericarp.

Plant hormones play different biological roles in the anthocyanin biosynthesis. The applications of exogenous plant hormones can regulate the synthesis of anthocyanins. For example, NAA (1-Naphthaleneacetic acid) treatment can alter ethylene production, which in turn induces ripening in sweet cherry and enhanced anthocyanin production, possibly through ABA metabolism [69]. Exogenous treatment with S-ABA promoted accumulation of total anthocyanins in the skin of berries and increased the expressions of *CHI*, *F3H*, *DFR*, *UFGT*, *VvMYBA*1 and *VvMYBA2* in the seedless grape cultivar [70]. Cytokinin treatments can inhibit the expression of MdMYBL2, a negative regulator of anthocyanin biosynthesis, but upregulated the expression of *MdDFR*, *MdUFGT*, *MdMYB10* and *MdbHLH3* [71]. While the regulation of anthocyanin biosynthesis by plant hormones may not be a simple process, but a complex network of interactions with other environmental factors. Therefore, the studies between plant hormones and anthocyanin biosynthesis are still needed. Thoroughly elucidation of the molecular regulation of anthocyanin biosynthesis will provide guidance for molecular breeding to select new varieties rich in anthocyanin.

## Conclusions

Collectively, the DEGs and DAFs involved in anthocyanin metabolism were analyzed by combined metabolomic and transcriptomic analysis. Flavonoid compounds of chalcone, flavonol, proanthocyanidin and anthocyanidin were the most differentially accumulated in Hengzi151. The expressions of some structural genes were prominently activated in Hengzi151, such as *PAL*, *CAD*, *CHS*, *FLS*, *F3H*, *F3′H*, *DFR*, *ANS*, *ANR* and *UFGT* genes. *MYB*, *bHLH*, *WD40* genes and other TF genes, such as *bZip*, *NAC*, *WRKY*, *MADS* were also identified. Some genes related to hormone synthetic and signaling pathway involved in regulating anthocyanin biosynthesis also have been identified. The discovery revealed the pathways and key genes of anthocyanin biosynthesis in wheat, which provided a reference for further study on anthocyanin biosynthesis.

## Supplementary Information

Supplementary Material 1. Fig. S1 Correlation analysis according to metabolome and transcriptome data. The upper right corner represents the correlation of the transcriptomes, the upper left corner stands for metabolomes.

Supplementary Material 2. Fig. S2 Cluster analysis of DAFs between Hengzi151 and Shiluan02-1 mapped to flavonoid biosynthetic pathway (taes00941, a), anthocyanin biosynthetic pathway (taes00942, b), and flavone and flavonol biosynthetic pathway (taes00944, c).

Supplementary Material 3. Fig. S3 The quadrant diagram representing the association of correlated (R^2^ > 0.9) genes and flavonoid metabolites between Shiluan02-1 and Hengzi151.

Supplementary Material 4. Fig. S4 An integrated O2PLS analysis of the transcriptome and metabolome datasets, the top 15 metabolites substantially affected the transcriptome and the top 15 genes in transcriptome strongly influenced the metabolome. The X-axis means the loading value.

Supplementary Material 5. Fig. S5 Connection network between DEGs and DAFs in Hengzi151 vs. Shiluan02-1 comparison in the taes00942 (a) and taes00944 (b) pathways.

Supplementary Material 6. Fig. S6 The DEGs in signal transduction pathways of hormones.

Supplementary Material 7. Table S1: The information of metabolome.

Supplementary Material 8. Table S2: The differentially expressed flavonoids compounds between Shiluan02-1 and Hengzi151.

Supplementary Material 9. Table S3: The differentially expressed MYB, bHLH and WD40 genes identified between Shiluan02-1 and Hengzi151.

Supplementary Material 10. Table S4: The other identified differentially expressed transcription factors between Shiluan02-1 and Hengzi151.

Supplementary Material 11. Table S5: The DEGs of PAL, CHI, CHS and F3H related to flavonoids.

Supplementary Material 12. Table S6: Sequences of specific primers for qRT-PCR.

## Data Availability

The sequencing data was deposited in NCBI database under accession number: PRJNA984599.
