# Supplementary figures and images for: Integrated metabolomic and transcriptomic analysis of anthocyanin metabolism in wheat pericarp
Source: BMC Genom Data. 2025 Jan 13;26:3. doi: 10.1186/s12863-024-01294-y (PMC11727400; doi:10.1186/s12863-024-01294-y)

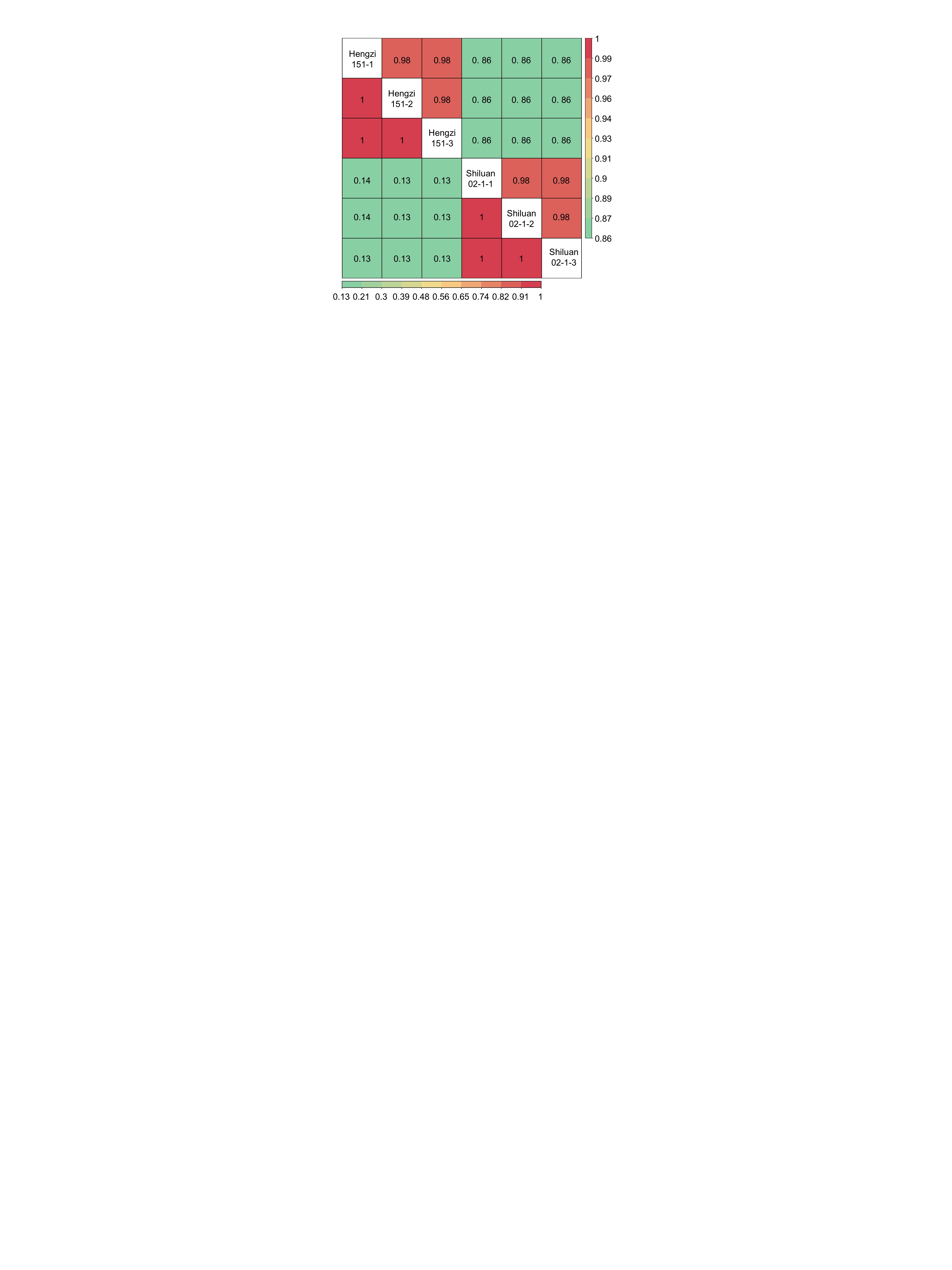

Supplement: Supplementary file 1 — Supplementary Material 1. Fig. S1 Correlation analysis according to metabolome and transcriptome data. The upper right corner represents the correlation of the transcriptomes, the upper left corner stands for metabolomes. [file 12863_2024_1294_MOESM1_ESM.jpg]

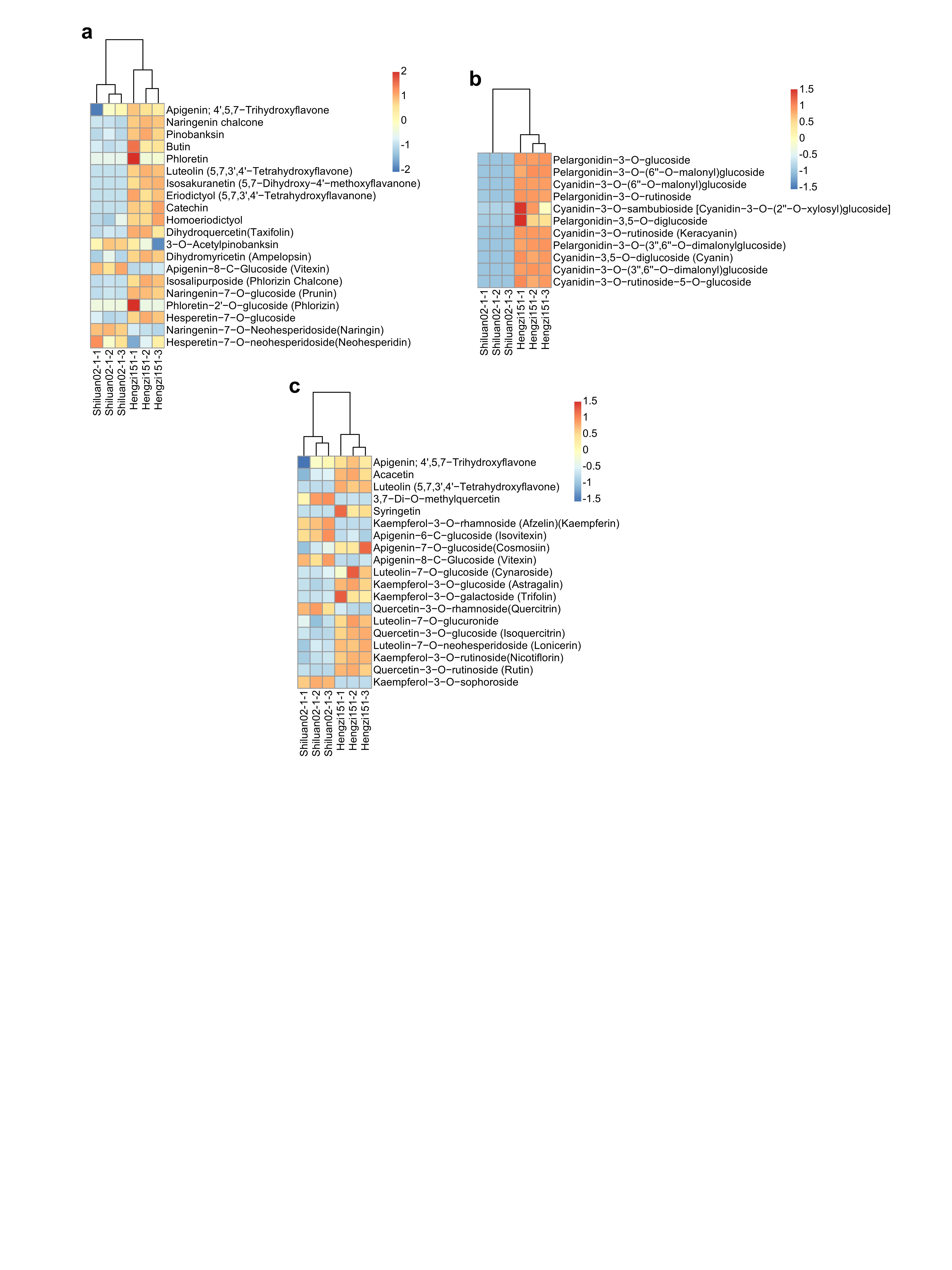

Supplement: Supplementary file 2 — Supplementary Material 2. Fig. S2 Cluster analysis of DAFs between Hengzi151 and Shiluan02-1 mapped to flavonoid biosynthetic pathway (taes00941, a), anthocyanin biosynthetic pathway (taes00942, b), and flavone and flavonol biosynthetic pathway (taes00944, c). [file 12863_2024_1294_MOESM2_ESM.jpg]

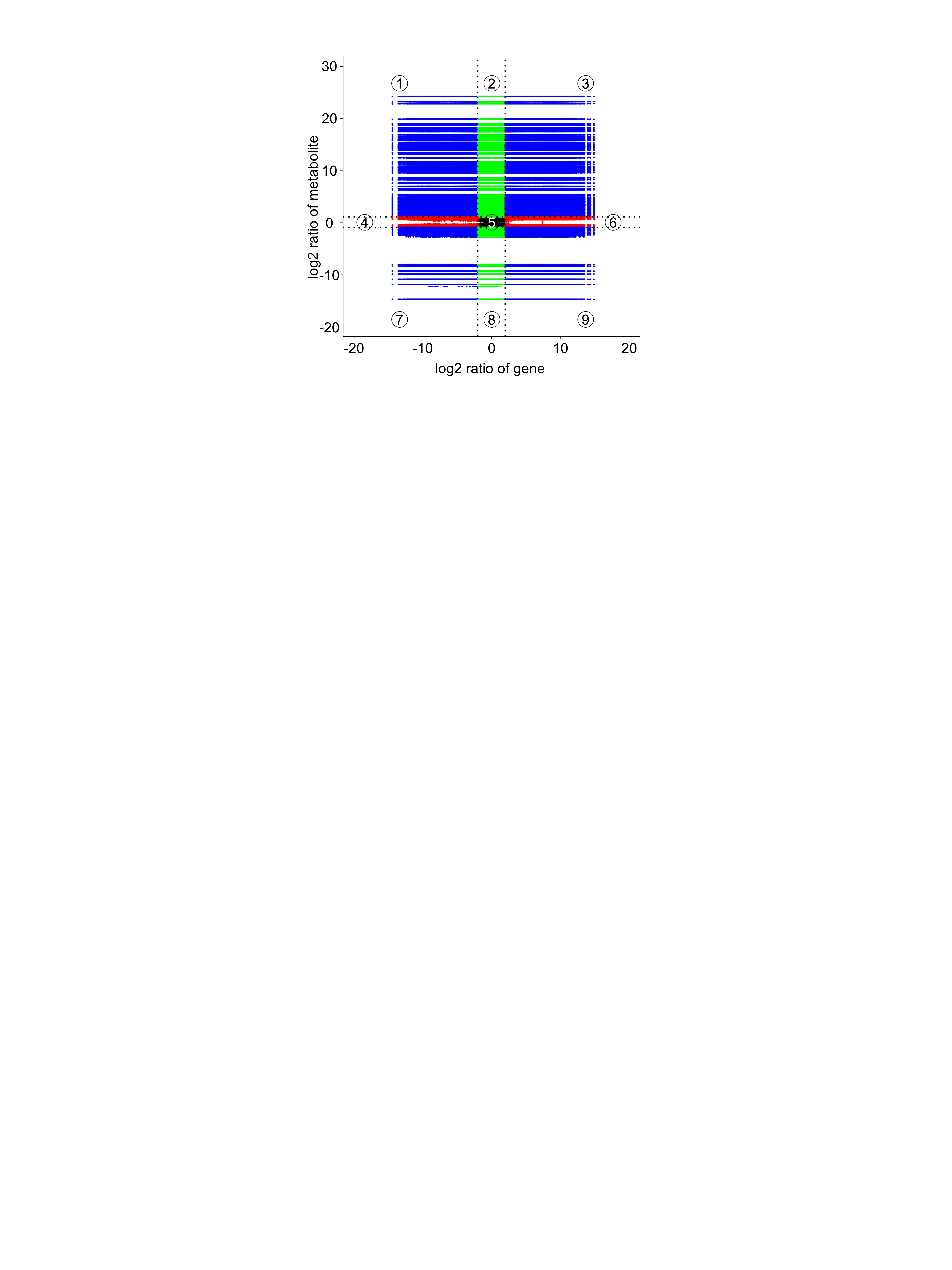

Supplement: Supplementary file 3 — Supplementary Material 3. Fig. S3 The quadrant diagram representing the association of correlated (R2 > 0.9) genes and flavonoid metabolites between Shiluan02-1 and Hengzi151. [file 12863_2024_1294_MOESM3_ESM.jpg]

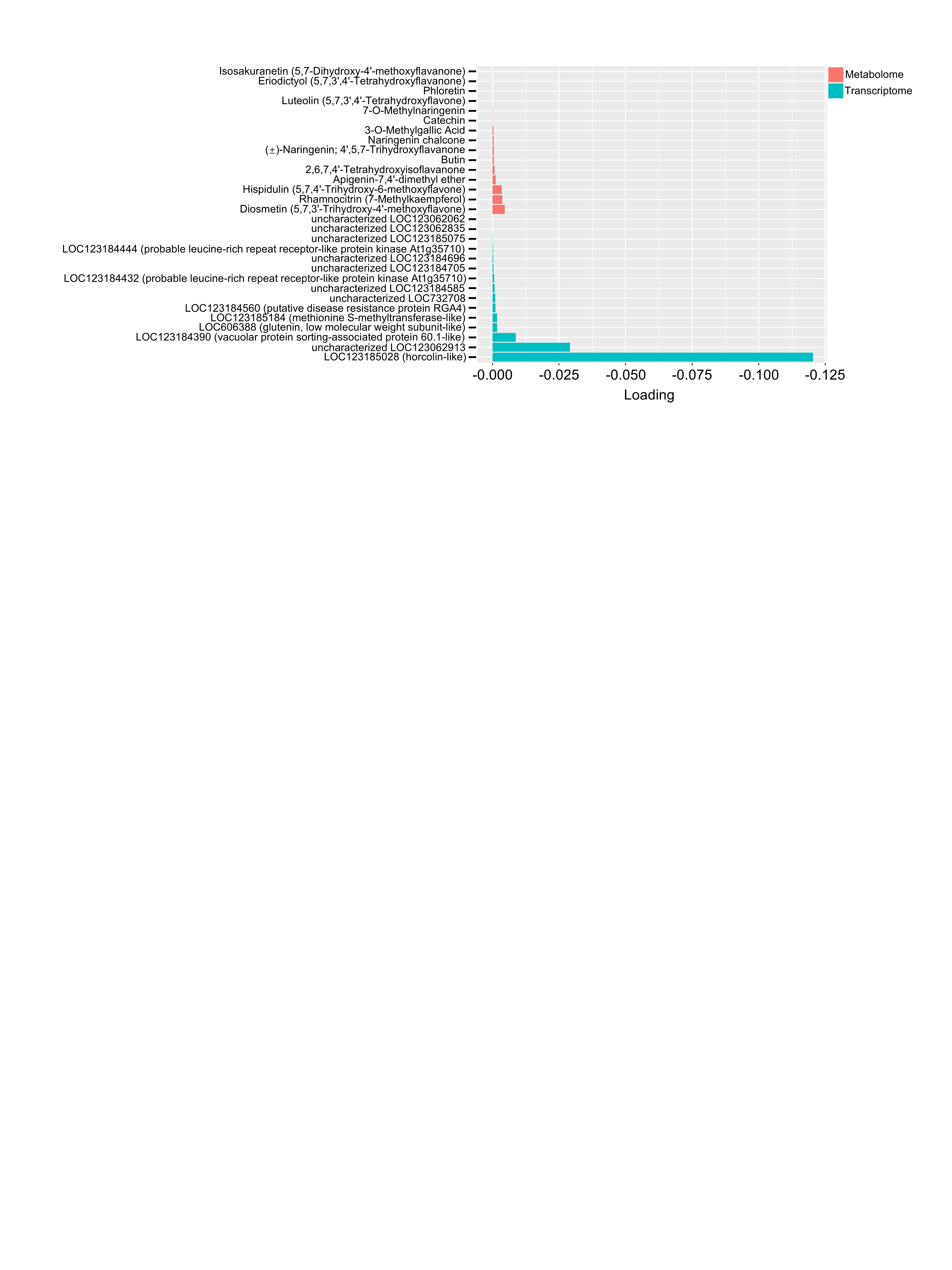

Supplement: Supplementary file 4 — Supplementary Material 4. Fig. S4 An integrated O2PLS analysis of the transcriptome and metabolome datasets, the top 15 metabolites substantially affected the transcriptome and the top 15 genes in transcriptome strongly influenced the metabolome. The X-axis means the loading value. [file 12863_2024_1294_MOESM4_ESM.jpg]

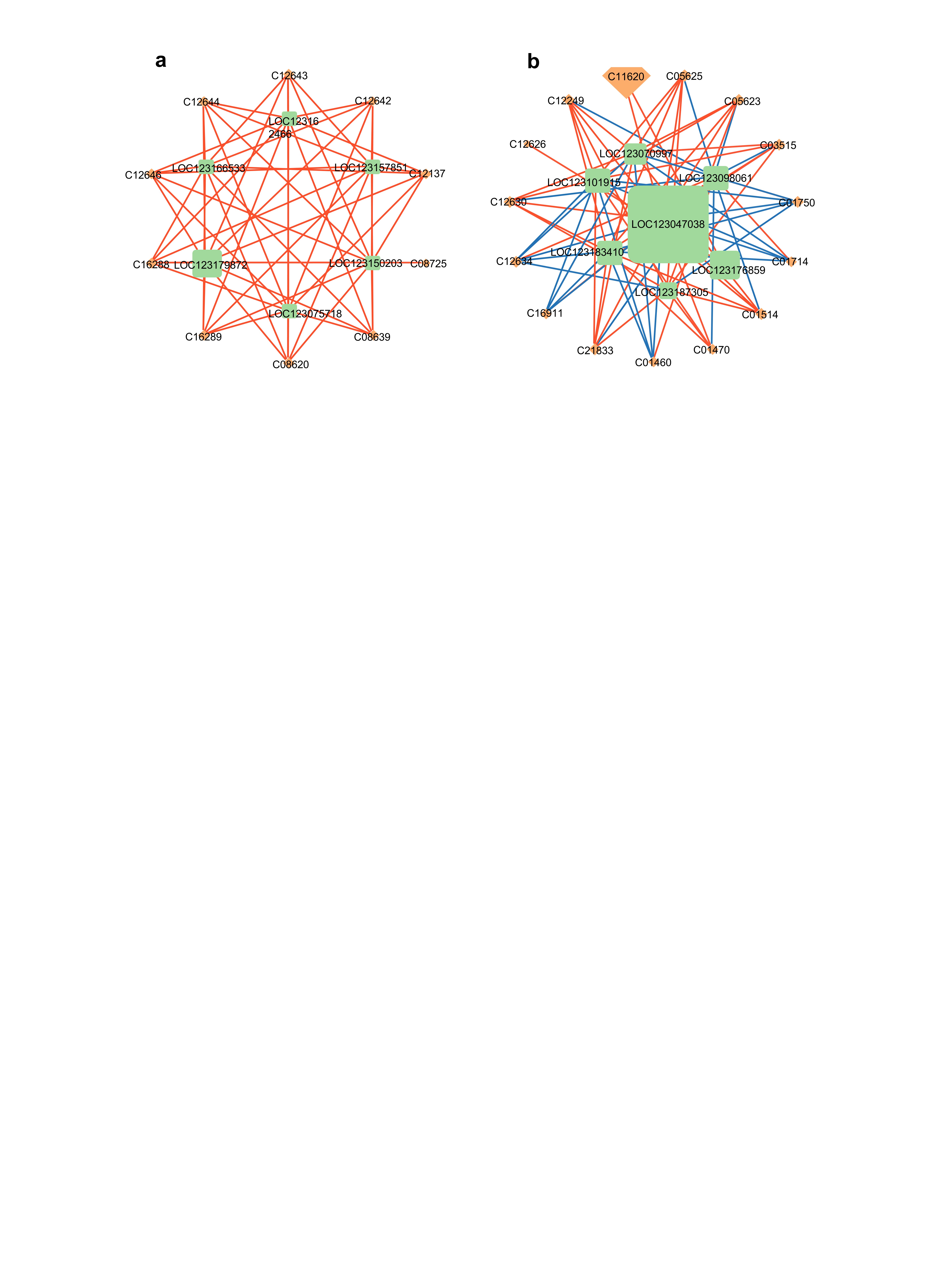

Supplement: Supplementary file 5 — Supplementary Material 5. Fig. S5 Connection network between DEGs and DAFs in Hengzi151 vs. Shiluan02-1 comparison in the taes00942 (a) and taes00944 (b) pathways. [file 12863_2024_1294_MOESM5_ESM.jpg]

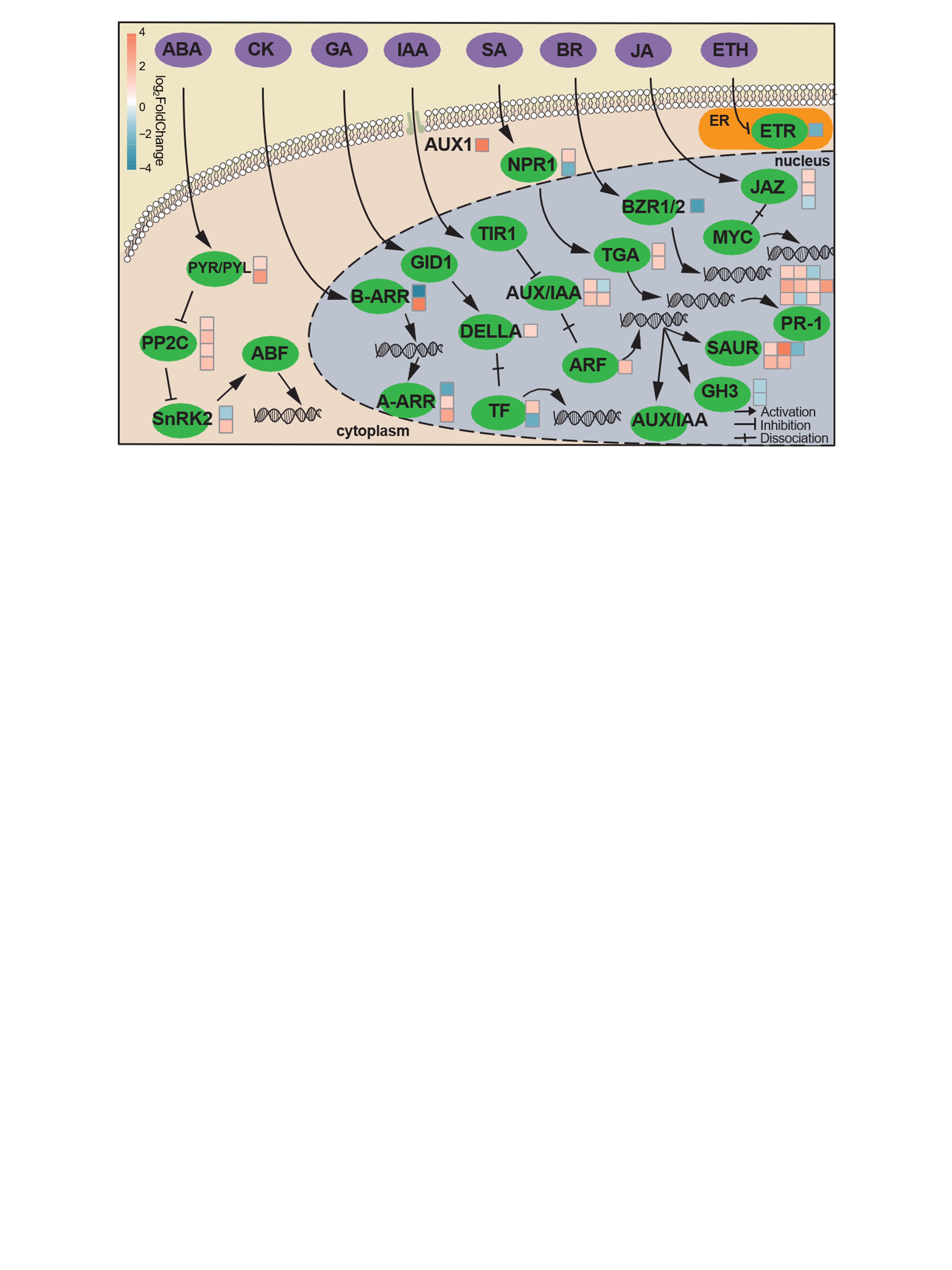

Supplement: Supplementary file 6 — Supplementary Material 6. Fig. S6 The DEGs in signal transduction pathways of hormones. [file 12863_2024_1294_MOESM6_ESM.jpg]
